# Characterizing the Late Pleistocene MSA Lithic Technology of Sibudu, KwaZulu-Natal, South Africa

**DOI:** 10.1371/journal.pone.0098359

**Published:** 2014-05-30

**Authors:** Manuel Will, Gregor D. Bader, Nicholas J. Conard

**Affiliations:** 1 Department of Early Prehistory and Quaternary Ecology, University of Tübingen, Schloss Hohentübingen, Tübingen, Germany; 2 Senckenberg Center for Human Evolution and Paleoecology, University of Tübingen, Schloss Hohentübingen, Tübingen, Germany; University of Oxford, United Kingdom

## Abstract

Studies of the African Middle Stone Age (MSA) have become central for defining the cultural adaptations that accompanied the evolution of modern humans. While much of recent research in South Africa has focused on the Still Bay and Howiesons Poort (HP), periods following these technocomplexes were often neglected. Here we examine lithic assemblages from Sibudu that post-date the HP to further the understanding of MSA cultural variability during the Late Pleistocene. Sibudu preserves an exceptionally thick, rich, and high-resolution archaeological sequence that dates to ∼58 ka, which has recently been proposed as type assemblage for the “Sibudan”. This study presents a detailed analysis of the six uppermost lithic assemblages from these deposits (BM-BSP) that we excavated from 2011–2013. We define the key elements of the lithic technology and compare our findings to other assemblages post-dating the HP. The six lithic assemblages provide a distinct and robust cultural signal, closely resembling each other in various technological, techno-functional, techno-economic, and typological characteristics. These results refute assertions that modern humans living after the HP possessed an unstructured and unsophisticated MSA lithic technology. While we observed several parallels with other contemporaneous MSA sites, particularly in the eastern part of southern Africa, the lithic assemblages at Sibudu demonstrate a distinct and so far unique combination of techno-typological traits. Our findings support the use of the Sibudan to help structuring this part of the southern African MSA and emphasize the need for further research to identify the spatial and temporal extent of this proposed cultural unit.

## Introduction

Recent archaeological, palaeoanthropological and genetic research demonstrates that modern humans evolved on the African continent. Fossils of modern humans date back as far as 200.000 years ago ( = 200 ka), and starting from Africa *Homo sapiens* dispersed to the rest of the world [1]–[6]. Studies in the African Middle Stone Age (MSA), which dates between ca. 300 and 30 ka, have focused on the biological and behavioral evolution of our species, as well as the geographic expansion of modern humans. The MSA of southern Africa plays a central role in these questions due to its long research history and the wealth of excavated sites [7]–[9]. Most importantly, southern African MSA sites including Klasies River [10], [11], Blombos [12], [13], Pinnacle Point 13B [14], [15], Sibudu [16], [17], and Diepkloof [18]–[20] provide a long and well-dated chrono-cultural framework.

With documentation of the biological origin of *Homo sapiens* in Africa [1]–[4], researchers shifted their focus to the MSA, which had been previously neglected, to examine the nature and tempo of cultural change in early modern humans. Since the late 1990s, archaeological finds in the southern African MSA with unexpectedly early dates led researchers to rethink the evolution of modern human behavior. These finds include among others: abstract depictions on ochre and ostrich eggshell [21]–[24], ochre processing kits [13], personal ornaments [25], [26], bone artifacts [27], [28], heat treated artifacts [29], and potentially bow and arrow technology [30]. Due to these discoveries, the African continent and particularly southern Africa has become the center of attention for studying the cultural evolution of *Homo sapiens* [1], [5], [31] (but see [32]–[34]).

Many of these early complex elements of the material culture were observed in two sub-stages of the southern African MSA, the Still Bay (SB) and Howiesons Poort (HP). Backed tools and laminar technology characterize the HP, whereas bifacial technology with foliate points mark the SB [35]–[39]. Scholars often consider these cultural units as indicating advanced cognition and sophisticated socio-economic behaviors of their makers. This view has resulted in a strong research emphasis on the SB and HP [5], [40]–[45]. Some researchers even associate the innovative technological and socio-economic aspects of the SB and HP with subsequent dispersals of modern humans to Eurasia (e.g. [5], [46]).

While research has focused on the supposedly unique aspects of the SB and HP, earlier and later periods of the MSA were often considered as unsophisticated, less innovative or conventional in their technology. In this view, the SB and HP represent two short-lived but culturally advanced episodes preceded and followed by less behaviorally sophisticated phases. Based on this reasoning, some scholars invoke a model of discontinuous cultural evolution in modern humans in which complex material culture appears and disappears abruptly in the South African MSA [41], [43], [47]–[51]. Although ecological causes are sometimes cited (e.g. [47], [51]), most of the proponents of these ideas call upon demographic collapses to explain their model. As a consequence of this purported depopulation, smaller isolated groups of people lost traditions that were previously shared with other groups over large areas (e.g. [41], [50]).

These views have increasingly attracted criticism. Some scholars argue that the proposed model of cultural evolution is overly simplistic [52], [53]. Moreover, the current archaeological evidence contradicts this theory: many SB and HP localities such as Diepkloof, Sibudu or Klasies River were not abandoned by the inhabitants afterwards. Instead, people occupied these sites continuously without evidence for stratigraphic hiatuses. Phases of occupation that follow the HP sometimes even exhibit higher intensities of settlement, such as at Sibudu. Additionally, recent synthetic research has found that more sites existed at ∼58 ka than during the SB phase [54]–[57], although differences in settlement systems, taphonomy and discovery biases might influence this measure. Current studies on lithic assemblages from the SB and HP have also documented a higher degree of temporal and regional variability than acknowledged before [35], [39], [58]–[62]. At Diepkloof, researchers have argued that both SB and HP occupations date earlier and last longer than at other MSA localities in southern Africa [20]. Based on current evidence, regional and temporal variation occur in all periods of the MSA and the number and occupation intensities of sites post-dating the HP appear to refute hypotheses favoring demographic collapses following this technocomplex.

The focus on the SB and HP remains a problem facing current research on technological variability during the southern African MSA. This emphasis has resulted in a lack of detailed studies for other phases of the MSA in an otherwise well-studied region (see [19], [35], [39], [52], [54], [60], [63], [64]). Hence, assemblages from these periods are frequently attributed to informal stages such as “post-HP” or “pre-SB”. Considering this research bias, it comes as no surprise that some scholars consider lithic assemblages after the SB and HP as technologically rudimentary, unsophisticated, or a return to a conventional “pre-SB” MSA [41], [47], [50], [65]–[67]. Yet, in order to track technological change in the southern African MSA, all of its phases must be studied with the same intensity.

### The “post-HP” of Southern Africa and at Sibudu

Regarding the later part of the southern African MSA, lithic assemblages that succeed the HP and fall within MIS 3 comprise the so-called “post-HP” [11], [68], “MSA 3” [8] or “MSA III” [10]. At present, these labels act as catch-all categories with little scientific value [54], [63], [69], [70]. For instance, Wadley ([69], p. 2404) summarizes the current view of the “post-HP” as being poorly understood while at the same time regarded as “dark ages” that followed the HP. Even so, many sites from this time period exist in southern Africa, such as Apollo 11, Border Cave, Diepkloof, Klasies River, Klein Kliphuis, Melikane, Sibudu, Sehonghong and Umhlatuzana (see [9], [54], [71]). They include localities with ephemeral settlements but also with thick occupation sequences (e.g. Sibudu, ca. 1.5 m from ∼58–38 ka [68], Klasies River, ca. 1.2 m at ∼58 ka [60]).

Finer subdivision of the MSA that follows the HP, covering a period of approximately 30 ka, have been made primarily at sites that feature long sequences from this time span. At Sibudu, for instance, Wadley and Jacobs [68] distinguish the informal phases “post-HP” (∼58 ka), “late MSA” (∼48 ka), and “final MSA” (∼38 ka). These informal terms, however, have not been applied by other researchers in a uniform manner. In most recent publications, the term “post-HP” is used to address the earlier phases of MIS 3 (ca. 58–40 ka; including “late MSA” assemblages) and “final MSA” – with hollow-based points as characteristic tool forms in KwaZulu-Natal – to denote the following period that ends with the onset of the LSA [9], [39], [57], [64], [71].

In terms of their geographical distribution, MSA sites postdating the HP occur throughout southern Africa and can be found in various climatic and environmental contexts (see [9], [54], [71]). A decline in the number and intensity of occupations after the HP in the Western Cape, especially between 50–25 ka (e.g. [19], [59], [71]), has sometimes been interpreted as indicating low population densities during MIS 3 in southern Africa (e.g. [41], [46], [48], [72], but see [54]). These observations, however, do not correspond to the pattern in the eastern part of southern Africa. Here, the number of sites increases and several localities with thick and rich occupation sequences, such as Umhlatuzana [73], [74] or Sibudu [57], [63], [68], occur during this period (see also [54], [71] for discussion and references).

Scholars defined the MSA lithic assemblages that follow the HP for the most part on the basis of what they lack, such as bifacial points or backed pieces, instead of what they contain (see [57], [63]). The only unifying characteristics frequently cited for the “post-HP” are a greater variety of flake tools and numerous unifacial points that replace backed artifacts as the principal tool category (e.g. [9], [39], [60], [71]). In our view, the “informal” or “conventional” MSA character that is often attributed to assemblages following the HP derives from a combination of several factors. First, they reflect a wide range of assemblages from different chronological, environmental and techno-economic contexts. Second, the lithic assemblages are often poorly studied and poorly published. Additionally, scholars have frequently mentioned the (near-) absence of engravings, ornaments or worked bone for this period (e.g. [5], [39], [40], [54]). While some of these elements of the material culture occur exclusively in the HP (e.g. engraved ostrich eggshell [23], [75]) and their quantity is much higher, assemblages of the “post-HP” in southern Africa have also provided worked bone [28], [76], potential engravings on ochre [77] and other elements of complex behaviour (see below).

**Figure 1:**
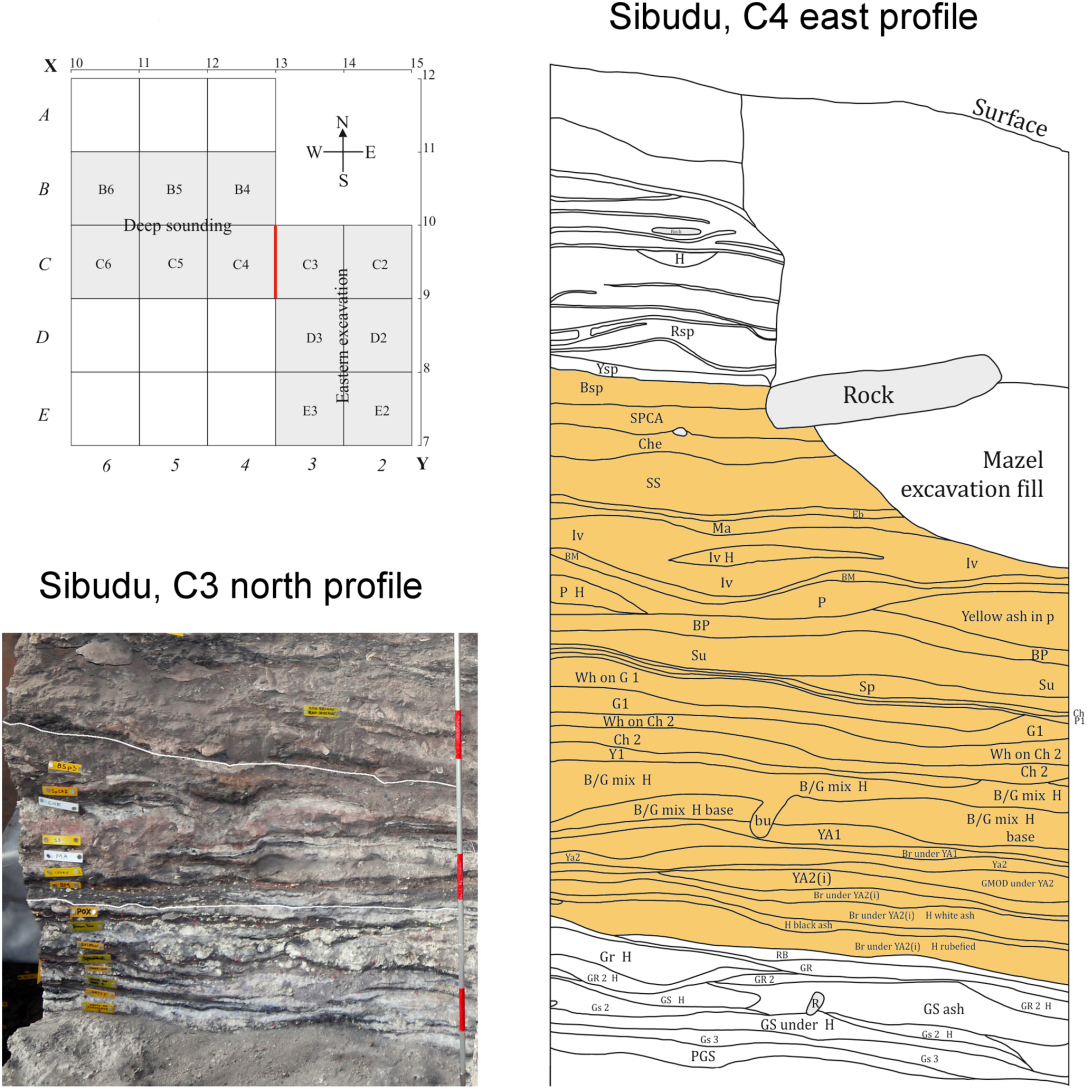
Excavation area and stratigraphic sections of the “post-HP” sequence from Sibudu. Upper left: Excavation grid. The lithic assemblages from the Sibudan come from the “Eastern Excavation”. Right: Sketch of the stratigraphic section of the eastern profile (C4, after Wadley). The complete “post-HP” sequence is highlighted in orange (layers BSP-BR Under YA2). Bottom left: Photograph depicting the stratigraphic section of the northern profile (C3) during excavations in 2013. The white lines mark the seven uppermost layers of the “post-HP”, or Sibudan, sequence from the top of BSP until the bottom of BM. Note the very fine lamination of archaeological layers in different colors caused by frequent combustion features (photograph by M. Will).

It is the main objective of this paper to help correct the research bias toward the HP and SB by providing new, detailed data on lithic assemblages that follow these technocomplexes. Our work concentrates on the archaeological site of Sibudu as it constitutes a promising candidate to study the period following the HP. The “post-HP” sequence at Sibudu is approximately one meter thick with more than 30 individual archaeological layers [68]. These finely laminated horizons provide the best stratigraphic record of this period known anywhere on the sub-continent (Figure 1). Archaeological layers at the top and base of this thick sequence have been dated to ∼58 ka, providing an exceptionally high temporal resolution. The whole “post-HP” sequence might have accumulated over only a few centuries or millenia [40], [57], [68].

Recent research on the “post-HP” sequence of Sibudu contradicts notions of large-scale population collapses after the HP. These studies also provide ample evidence for advanced technological behaviors of modern human populations living at Sibudu during this period. The sequence that follows the HP (<60 ka) exhibits burning events that are frequently stacked, indicating that people made repeated use of hearths and settled more intensively at the site after the HP [70], [78]–[82]. Results from dating and sediment micromorphology support this assertion in showing a higher rate of anthropogenic sedimentation and find densities in the these layers [63], [68], [83]. Geoarchaeological analyses document that the inhabitants constructed bedding made from sedges in the “pre-SB”, HP, “final MSA” and “post-HP” layers [80], [83], [84]. The more frequent occurrence of bedding constructions, burning and other forms of site use and maintenance during the “post-HP” suggests intensified occupations and a change in domestic organization [80], [83]. Just as during the SB and HP, people produced ochre powder on-site during the “post-HP” [69], [77] and used it as part of a compound adhesive for hafting stone tools, indicating advanced mental capacities and technical skill [85]–[90]. A particular phenomenon of the “post-HP” layers are large patches of ground ochre on the cemented ashes of burnt-out hearths. Wadley [69] argues that these cemented ashes served as work surfaces for the production of ochre powder, suggesting an especially extensive use of this raw material. Bone tools, often cited as markers of cultural complexity [27], [91], [92], occur in the “Pre-SB”, SB, HP and “post-HP” assemblages. The “post-HP” yielded two notched pieces, one smoother, one splintered piece, and one pressure flaker [28], [76].

On the basis of these features and an analysis of the highly structured and characteristic tool assemblages, Conard, Porraz and Wadley [63] recently proposed Sibudu as the type locality of a new sub-unit of the MSA, the “Sibudan” [63] which is not identical with the “Sibudu technocomplex” proposed by Lombard *et al.* [9]. They ([63], p. 181) justified the naming of a new sub-unit of the MSA on the basis that “informal terminology is untenable, because it implies that material cultural remains can be characterized by what they are not, rather than by their positive characteristics”. Conard *et al.* [63] distanced themselves from the informal “post-HP” and proposed the term Sibudan for the assemblages they studied, based on positive features. They stress that the Sibudan is not intended as a one-to-one equivalent of the “post-HP” of southern Africa, which would simply be replacing one label with another. Instead, the term is used to organize the many excavated assemblages from Sibudu, with these high-quality lithic data providing a point of comparison for further research. Conard *et al.* ([63], p. 181) also emphasize “that defining a new cultural taxonomic unit is a process” and they recommend conducting additional research to evaluate the viability of this term. In conclusion, they proposed the Sibudan as an organizational unit that constitutes a first step towards the nomenclature for the cultural sequence after the HP. We thus regard the Sibudan as a cultural-taxonomic unit that needs better characterization and contextualization in order to test its utility.

While Conard *et al.* [63] studied the tool assemblages and proposed a working model to characterize them, complete data was not then available on other technological aspects of these lithic assemblages. Here, we present our findings from a detailed technological analysis which are crucial to define the key elements of the Sibudan lithic technology and evaluate its short-term diachronic variability. With this approach we intend to further the understanding of technological variation during the Late Pleistocene MSA of southern Africa and provide a high-resolution empirical basis for comparative work. We also investigate the utility of the Sibudan as a possible cultural-taxonomic unit to help organize the sequence after the HP, by comparing our findings with lithic assemblages from other localities of this time period.

## Materials and Methods

**Figure 2:**
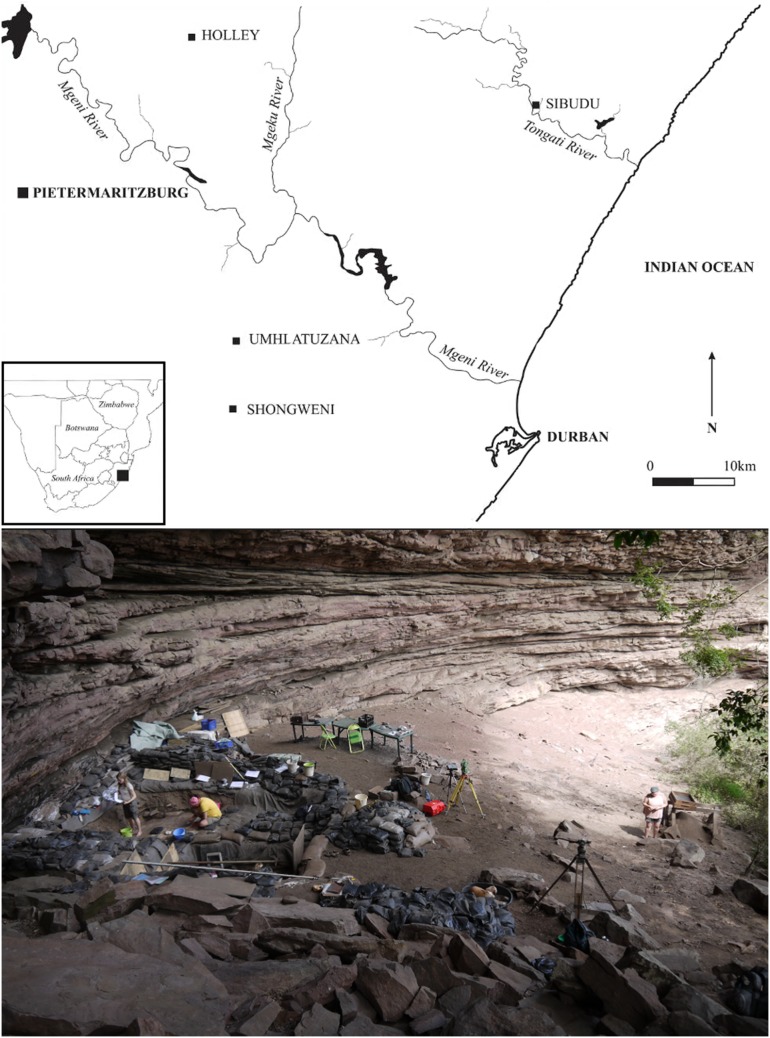
The archaeological site of Sibudu. Geographic location of Sibudu in KwaZulu-Natal (top, after [68]) and view on the excavation area within the rock shelter (bottom; photograph by M. Ecker).

The archaeological site of Sibudu is a large rock shelter situated above the Tongati River (also spelled “uThongathi”) in KwaZulu-Natal approximately 40 km north of Durban and 15 km from the Indian Ocean (Figure 2). The locality has yielded a rich archaeological sequence with deposits that span a time range of >75–37 ka [17], [68], [70], [78]. Sibudu is one of the few sites in South Africa that has yielded evidence for both SB and HP occupations, as well as the periods before and after [40], [63], [78]. The long-term excavations by L. Wadley provide a sound stratigraphic framework [17], [68]. The archaeological layers discussed here are almost completely anthropogenic, show little post-depositional disturbance and feature good organic preservation [70], [79], [80]. New field work at Sibudu has been carried out by a team of the University of Tübingen under the direction of N. Conard since 2011, building on the previous excavations by L. Wadley. The research permit to conduct archaeological excavations at Sibudu is issued under the KwaZulu-Natal heritage Act No. 4 of 2008 by Amafa AkwaZulu-Natali and is valid until December 2017. The permit holder is Nicholas Conard of the University of Tübingen (permit number: REF: 0011/14; 2031CA 070). All recovered archaeological specimens are permanently stored at the KwaZulu-Natal Museum in Pietermaritzburg (South Africa, 237 Jabu Ndlovu Street).

During the excavations we adopted Wadleýs stratigraphic system and layer designations (see [68] Tab. 2) and added systematic 3D piece plots of all classes of archaeological materials with a total station to the field methods. In each quarter meter, excavation proceeded in 2–3 cm thick *Abträge* that followed the slope of the sediments and never crosscut geological strata. The maximum volume of one *Abtrag* was a 10-liter bucket of sediment. These *Abträge* constitute the smallest time unit we discern at Sibudu and sometimes equal defined archaeological strata. We chose archaeological layers as the units to analyze the lithic assemblages as they constitute the best basis for inter-assemblage comparisons.

For this study, we analyzed the lithic assemblages from the 7 uppermost layers BM-BSP of the “post-HP” sequence from an area of 6 m^2^ (ca. 1.5 m^3^ of sediment; Figure 1), that we excavated in three seasons between 2011–2013. The results for six of these assemblages are presented in the following, with one layer (SS) being excluded due to the low number of lithic artifacts (n<100). The assemblages contain a total of 59,390 stone artifacts, with 2,649 pieces >25 mm and 56,741 small debitage products <25 mm (Table 1). For a detailed characterization of the technology of these assemblages, we examined the procurement and use of lithic raw materials, investigated reduction sequences, evaluated the methods and techniques of reduction and performed typological and techno-functional analyses of tools.

**Table 1:** Distribution of single finds (>25 mm) and small debitage (<25 mm).

Layer	Single finds	Small debitage	Total lithics
BSP	822	13644	14466
SPCA	578	10019	10597
CHE	133	2792	2925
MA	178	4421	4599
IV	676	20389	21065
BM	262	5476	5738
Total	2649	56741	59390

We examined all stone artifacts >25 mm individually, combining attribute analysis and reduction sequence approaches. Attribute analysis quantifies the various traces on lithic artifacts that result from the knapping process and records metric traits in order to reconstruct technological behavior [93]–[97]. In addition to observations by hand lenses we sometimes used light microscopy. Our qualitative investigation follows the concept of *chaîne opératoires* [99]–[100] or reduction sequences [101]–[103]. This approach studies the methods of core reduction and the stages of lithic manufacture that people performed at the site. We also conducted quantitative analyses on samples of the small debitage products to calculate raw material proportions and frequencies of retouching activities.

As the method of core reduction constitutes an essential point in characterizing the technology of MSA people, and description of core types should be comparable between sites, we employed the unified taxonomy by Conard *et al.* [104]. We analyzed the tool inventories of the lithic assemblages with regards to typological, technological and techno-functional aspects. Although researchers have legitimately criticized the traditional typological approach to retouched artifacts [105]–[108], a list of defined tool types still provides a broad means of comparison between different sites and technocomplexes. We recorded tool types with a special recognition of the typology of the southern African MSA (*cf*. [109]–[112]). Most importantly, scholars in South Africa have defined “unifacial points” in a very broad sense which include a wide range of convergent and pointed forms with both marginal and invasive retouch. A unifacial point in this definition may be the equivalent of a convergent scraper, a marginally retouched Levallois point, or a triangular flake that was modified at the distal tip only [63], [111], [112].

**Figure 3:**
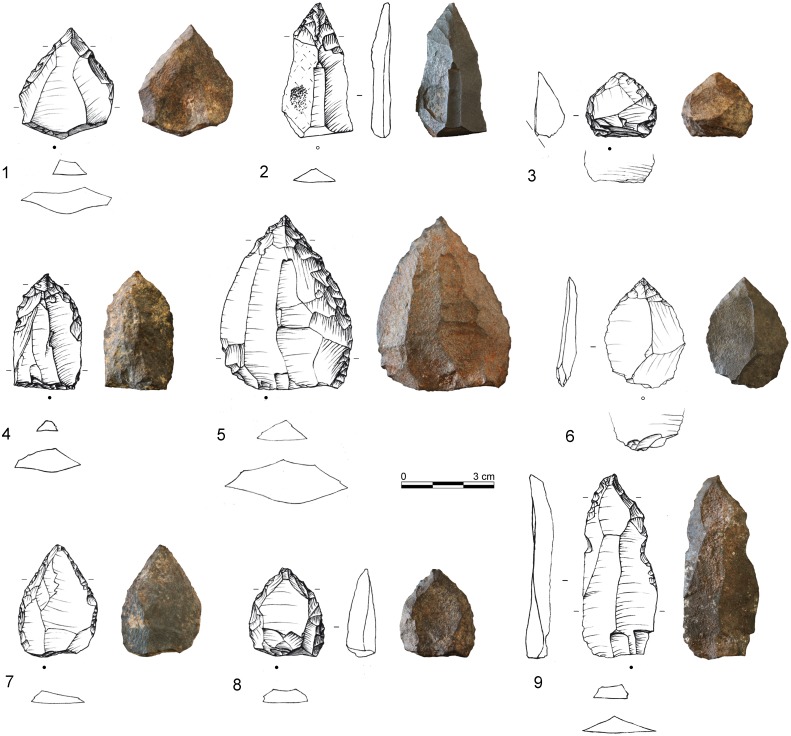
Examples of Tongatis from BM-BSP. 1: IV, dolerite, D3-532.1; 2: BSP, hornfels, E3-8.6; 3: IV, dolerite, C3-631.2; 4: BM, dolerite, E2-444; 5: IV, dolerite, E3-665; 6: BM, hornfels, E3-737; 7: IV, dolerite, D3-435; 8: IV, dolerite, E3-584; 9: BM, dolerite, D2-434. Drawings by F. Brodbeck and G. Porraz; photographs by G. Porraz. After [63] Fig. 7.

**Figure 4:**
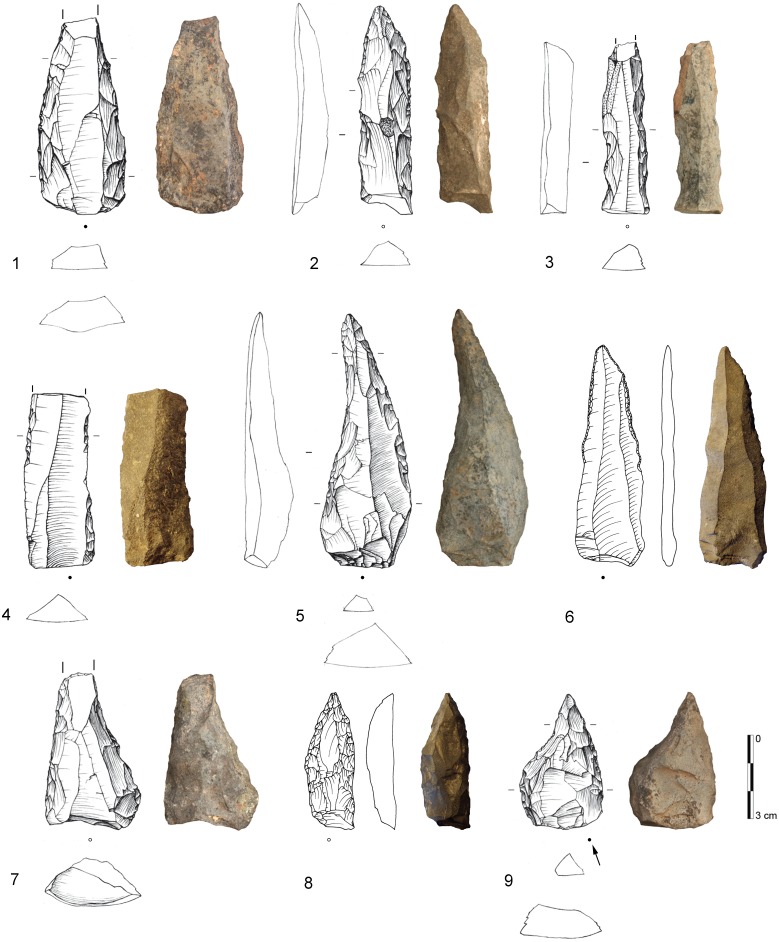
Examples of Ndwedwes from BM-BSP. 1: IV, dolerite, D3-460; 2: IV, dolerite, E2-322; 3: IV, dolerite, E3-521; 4: BSP, dolerite, D3-51; 5: IV, dolerite, D3-546; 6: BSP, hornfels, D2-77; 7: MA, dolerite, D2-293; 8: BSP, hornfels, D3-114; 9: SPCA, dolerite, E3-409. Drawings 1–5, 7, 9 by F. Brodbeck and G. Porraz; drawings 6 & 8 by M. Malina; photographs by G. Porraz. 1–3, 5, 7, 9 after [63] Fig. 11.

**Figure 5:**
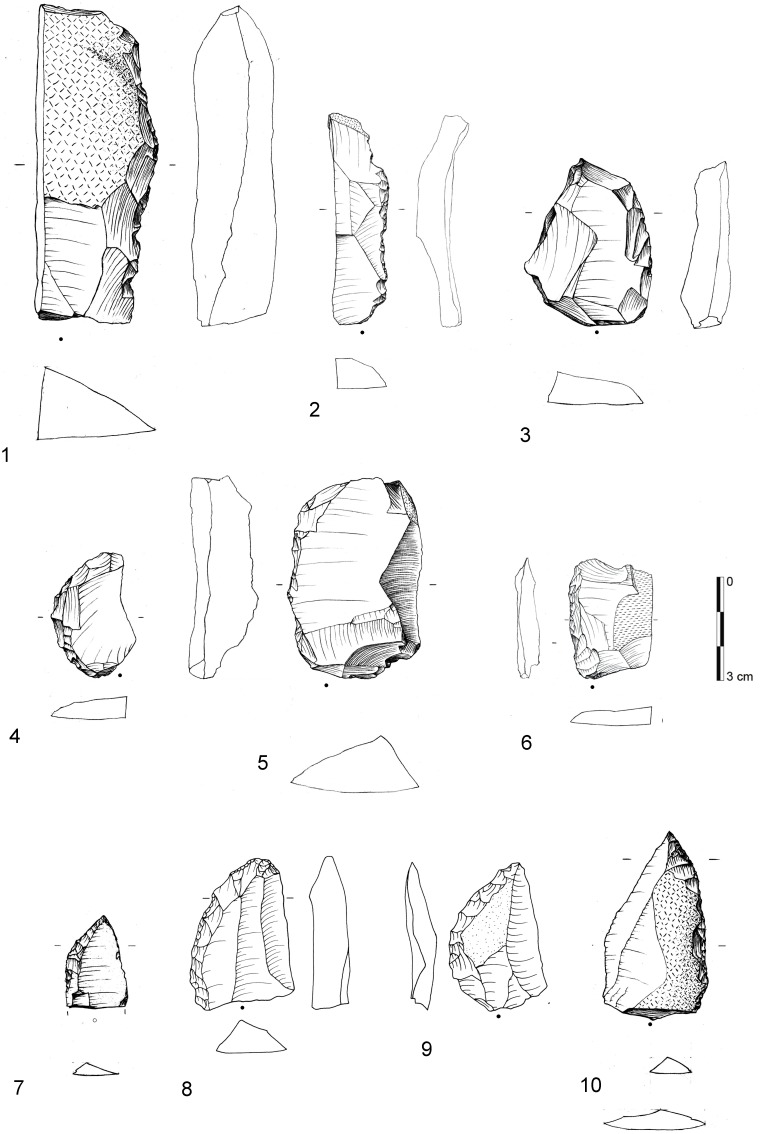
Naturally backed tools (1–6) and asymmetric convergent tools (7–10) from BM-BSP. 1: IV, dolerite, C3-392; 2: BSP, hornfels, C3-21; 3: IV, dolerite, E2-310; 4: IV, dolerite, E2-392; 5: BSP dolerite, D3-113; 6: SPCA, hornfels, E3-664; 7: BSP, hornfels, E3-38.2; 8: SPCA, dolerite, C2-237; 9: IV, dolerite, D3-371; 10: BSP, dolerite, E3-44. Drawings 1–7, 10 by F. Brodbeck and G. Porraz; drawings 8 & 9 by M. Malina. 1–5 after [63] Fig. 12.

Conard *et al.* [63] recently published a novel classification scheme for tools in the Sibudan based on a techno-functional method that differs from traditional typological (“type fossil”) approaches. This new procedure was devised, among other reasons, to organize assemblages rich in unifacial points, as the very broad definitions of unifacial points in South Africa obscure subtle morphological and metric differences. The new classification scheme rests mainly on an emphasis of the reduction and transformation of tool types that are usually treated as static entities [105], [107], [113]. In addition, they [63] employed a techno-functional approach (*sensu* [114]–[117]), which divides tools into a transformative, prehensile and intermediate part and studies the treatment of these portions separately. Upon these methods, Conard *et al.* [63] classified tools based on the identification of specific patterns of repetitive retouch on different parts of the tool which indicate formal and distinct retouching cycles. On these grounds, several tool classes and tool cycles were defined, including two categories that would usually be subsumed under the label unifacial points: “Tongatis” (Figure 3) and “Ndwedwes” (Figure 4). Conard *et al.* ([63]) provide further descriptions and depictions of these tool classes and their retouch cycles, including naturally backed tools (NBTs; Figure 5: 1–6). This new tool taxonomy presents a working model that needs to undergo critical appraisal with additional techno-functional, use wear and residue analyses.

In 2013, we recognized asymmetric convergent tools (ACT) as an independent tool class and retouch cycle among our enlarged sample of unifacial points of which the majority was originally classified as Tongatis. The main characteristic of ACTs is the eponym asymmetric and convergent distal end. It is formed by one convex retouched edge and one opposing straight edge which is frequently not retouched (Figure 5: 7–10). Additionally, most ACTs exhibit steeper retouch on the convex lateral, creating a blunt edge. The opposite straight edge features a sharp feathered termination. The cross-sections of ACTs are mostly asymmetric and often exhibit a thick ridge near the convexly retouched lateral edge. From our preliminary observations of the different varieties of these specimens and their reduction stages (n = 38), ACTs appears to change only at their initially unretouched working edge, where use-wear and edge damage accumulate continuously, thus decreasing the width of the piece during their tool cycle.

We analyzed flaking efficiency and reduction intensities for assemblages and individual raw materials as additional technological and techno-economic measures. Flaking efficiency measures the efficiency by which a knapping strategy converts a mass of stone into flake edge [118]–[120]. It is calculated for complete blanks by dividing edge length by mass. Higher values indicate a more efficient use of raw materials within assemblages. We use this measurement as it provides “an effective means of tracking technological change” ([120] p. 620). The reduction intensity of assemblages can have a strong influence on their technological and typological parameters. We thus examined it in two separate ways. For one, the ratio of blanks to cores provides a rough approximation. The higher the ratio, the more intense has an assemblage been reduced (e.g. [121]). Secondly, the intensity of core reduction can be measured by average core and flake length or thickness. Assemblages with shorter or thinner flakes and cores are more heavily reduced, assuming that knappers used nodules with consistent starting size [121], [122].

## Results

### Raw Material Procurement

**Figure 6:**
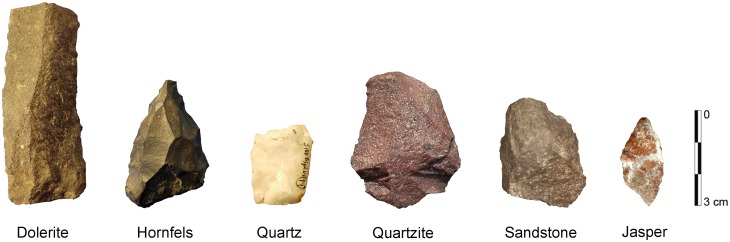
Selection of raw materials used by knappers in BM-BSP. Photographs by G.D. Bader.

Knappers at Sibudu used a variety of lithic raw materials. Results of previous studies [58], [123] suggest that they can be divided into two categories. The majority consists of local raw materials, including dolerite, quartzite, milky white quartz and sandstone. Non-local raw materials are mainly represented by hornfels, with rare pieces of jasper and crypto-crystalline silicates (CCS; Figure 6).

The local dolerite is an igneous granular-appearing rock that varies significantly in grain-size and mineral composition. In general, it is a hard, rough and homogeneous raw material. Dolerite occurs mainly as tabular slabs in sills and dykes. A dolerite intrusion into the sandstone cliff is located only a few hundred meters away from Sibudu. Further potential sources are a large number of dolerite dykes and sills in the near-by Dwyka tillite and the Pietermaritzburg Formation [123], [124]. The sandstone presumably derives from local resources, as the shelter itself is part of the Natal Group sandstones. However, people during the MSA also used sandstones that appear to be finer-grained than the shelter wall. The inhabitants of Sibudu collected most of the milky white quartz and quartzite from the Tongaati River where these raw materials still occur today [123]. Our own observations of frequent smoothed and rounded pebble cortex on these materials support this assertion.

Hornfels (metamorphosed shale) constitutes the finest-grained material used at Sibudu. It is dark-grey to black, dense, massive and has a high silica content. The hornfels shows favourable knapping characteristics and produces sharp but potentially fragile edges. Hornfels of the quality found in the MSA assemblages is not present in the direct vicinity of Sibudu today. The closest known outcrop of hornfels occurs in the Verulum area ∼15–20 km south of the site [123].

**Figure 7:**
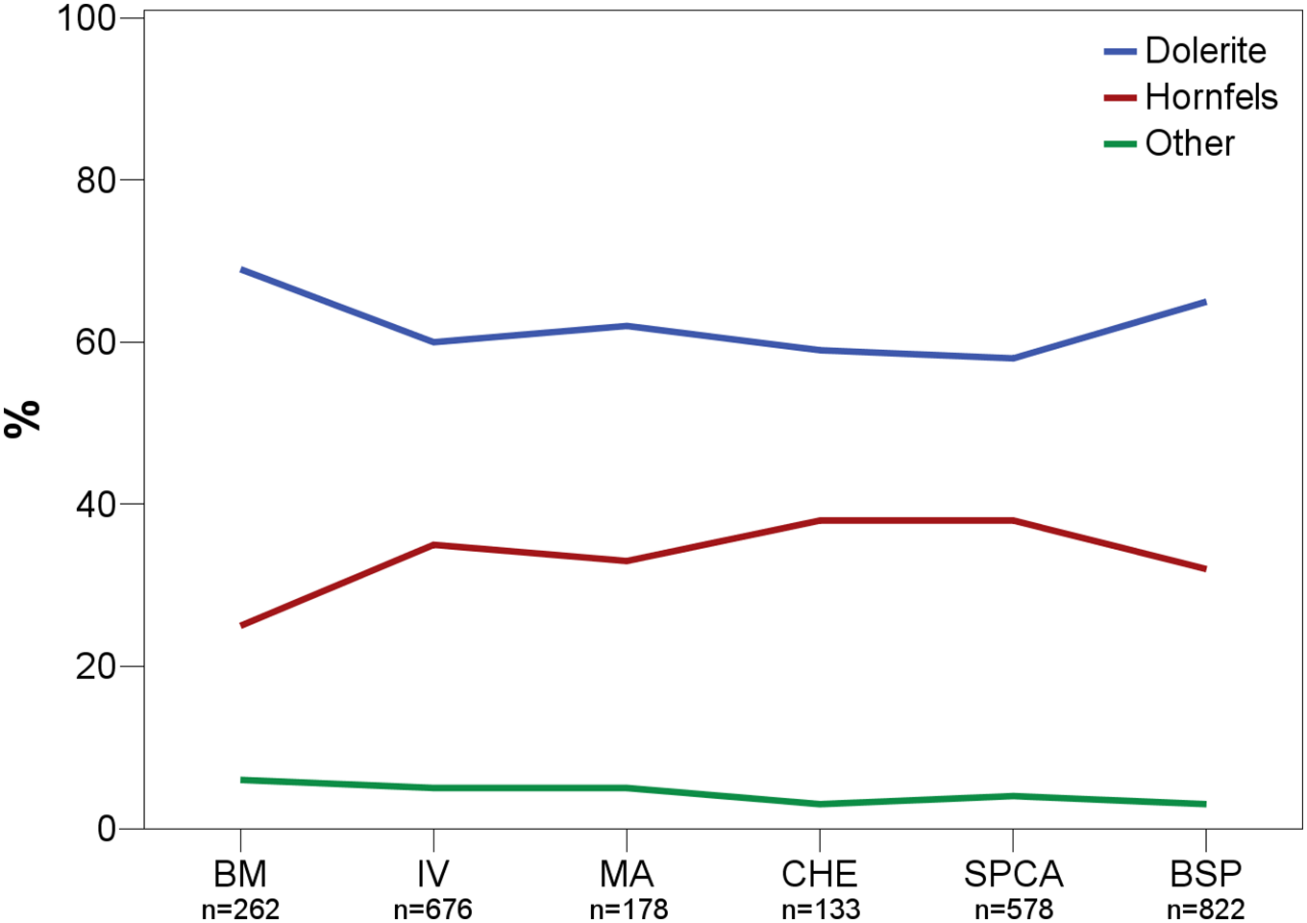
Percentual abundance of raw materials throughout BM-BSP. BM = oldest layer; BSP = youngest layer.

Knappers mainly used dolerite and hornfels for producing stone artifacts throughout BM-BSP, with a combined frequency of >93% for each assemblage (Table 2). Out of these two, dolerite dominates in all layers. Other raw materials like quartzite, quartz or sandstone never reach more than 5% abundance. CCS and jasper occur only in a few assemblages (CCS: BSP, SPCA; jasper: MA, IV) and in very small amounts (<1%). The inhabitants used principally the same range of raw materials throughout the sequence, and there is little diachronic variability in their abundance (Figure 7). The amount of dolerite as dominating raw material ranges between 58% (SPCA) and 69% (BM). The percentage of non-local hornfels varies between 25–38% and correlates negatively with the proportions for dolerite. The successive layers BM and IV exemplify this pattern, in which an increase in hornfels leads to a drop in dolerite and *vice versa*. Local raw materials always outnumber non-local ones, with the later accounting for roughly a third of the assemblages. In sum, we observe consistency in the choice and range of raw materials, including abundant import of non-local tool stones, with some temporal differences in the frequency of their use.

**Table 2:** Distribution of raw materials.

Layer	Dolerite	Hornfels	Sandstone	Quartzite	Quartz	Jasper	CCS	TOTAL
BSP	535 (65%)	262 (32%)	11 (1%)	8 (1%)	5 (1%)	-	1 (0%)	822
SPCA	333 (58%)	222 (38%)	11 (2%)	9 (2%)	2 (0%)	-	1 (0%)	578
CHE	79 (59%)	50 (38%)	4 (3%)	-	-	-	-	133
MA	111 (62%)	58 (33%)	4 (2%)	3 (2%)	1 (1%)	1 (0%)	-	178
IV	406 (60%)	235 (35%)	21 (3%)	6 (1%)	-	8 (1%)	-	676
BM	181 (69%)	66 (25%)	12 (5%)	2 (1%)	1 (0%)	-	-	262
Total	1645 (62%)	893 (34%)	63 (3%)	28 (1%)	9 (0%)	9 (0%)	2 (0%)	2649

Rounded percentages are given in brackets.

### Technological Aspects

#### Debitage analysis

**Figure 8:**
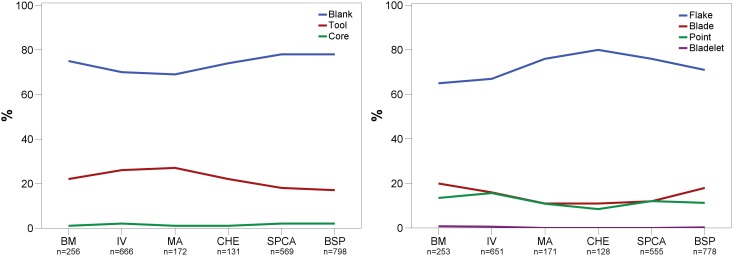
Frequencies of the main debitage categories (left) and blank types (right) produced throughout BM-BSP. BM = oldest layer; BSP = youngest layer.

A quantitative analysis of debitage products demonstrates that unretouched blanks constitute the main category of stone artifacts in all layers (>69%; Table 3). Angular debris and especially cores (∼2%) are rare. The most remarkable feature of the assemblages is their extraordinarily high proportion of retouched lithics compared to other MSA sites which are often characterized by less than 2% tools (e.g. [11], [109]). Tools account for an average of 21% of the analyzed stone artifacts >25 mm. The percentage of retouched specimens ranges between 17% (BSP) and up to 27% (MA), showing a consistent signal of abundant retouching activities (Figure 8).

**Table 3:** Quantitative debitage analyses of the main lithic categories.

Layer	Blank	Tool	Core	Angulardebris	TOTAL
BSP	640 (78%)	139 (17%)	19 (2%)	24 (3%)	822
SPCA	453 (78%)	104 (18%)	12 (2%)	9 (2%)	578
CHE	99 (74%)	29 (22%)	3 (2%)	2 (2%)	133
MA	123 (69%)	48 (27%)	1 (1%)	6 (3%)	178
IV	473 (70%)	179 (26%)	14 (2%)	10 (2%)	676
BM	196 (75%)	57 (22%)	3 (1%)	6 (2%)	262
Total	1984 (75%)	556 (21%)	52 (2%)	57 (2%)	2649

Rounded percentages are given in brackets.

#### Blank production

**Figure 9:**
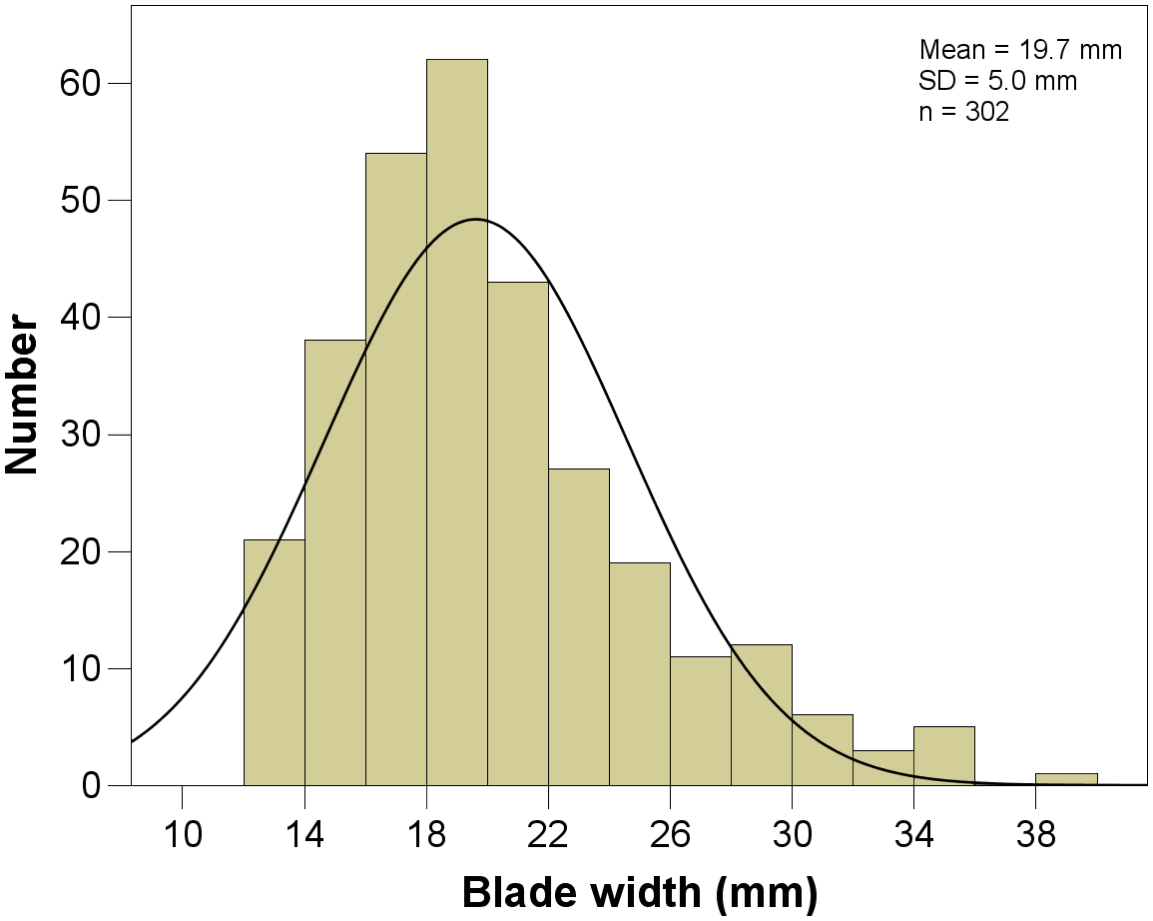
Distribution of blade widths (mm) from BM-BSP.

Flakes constitute the most frequent type of blanks produced (∼70%; Table 4). At the same time, blades and convergent flakes mark an important and persistent aspect of all assemblages (Figure 8). The proportion of blades varies between 11–20%, with convergent flakes being slightly less abundant (9–16%). There are clear sequences for the production of flakes, convergent flakes and blades, but not for bladelets. Most of the bladelets (n = 9; 0.4%) appear to be by-products of the laminar system that focussed on the manufacture of blades. The unimodal distribution of blade widths in all assemblages (Figure 9) and the lack of bladelet cores, with the exception of BSP and SPCA, support this interpretation (see *core reduction*). Throughout the sequence, a consistent proportion of about a third of the blanks is complete. Among the blank fragments, we found a particularly high proportion of longitudinal breaks (20–30%).

**Table 4:** Distribution of blank types.

Layer	Flake	Convergentflake	Blade	Bladelet
BSP	552 (71%)	88 (11%)	136 (18%)	2 (0%)
SPCA	418 (76%)	69 (12%)	68 (12%)	-
CHE	102 (80%)	12 (9%)	14 (11%)	-
MA	130 (76%)	22 (13%)	19 (11%)	-
IV	435 (67%)	105 (16%)	106 (16%)	5 (1%)
BM	165 (65%)	36 (14%)	50 (20%)	2 (1%)
Total^1^	1802 (71%)	332 (13%)	393 (16%)	9 (0%)

1 Including blank types of retouched artifacts.

Rounded percentages are given in brackets.

Knappers manufactured blanks that are relatively large. On average, (convergent) flakes are ∼40–42 mm long, occasionally exceeding 70 mm. The average length of blades is 48 mm with a width of 19 mm. Throughout the sequence, (convergent) flakes become increasingly larger. The oldest assemblage BM yields the smallest pieces, while the uppermost units SPCA and BSP demonstrate the largest ones. There is, however, no strong difference in their width or shape (length/width ratio). In contrast to these blank types, blades from all layers exhibit similar metric dimensions and length/width ratios of 2.5∶1. These observations suggest that the inhabitants followed a uniform approach to produce blades with standardized dimensions and shapes. The unimodal distribution of blade widths, clustering around 18–20 mm, supports this assertion (Figure 9).

#### Core reduction

**Figure 10:**
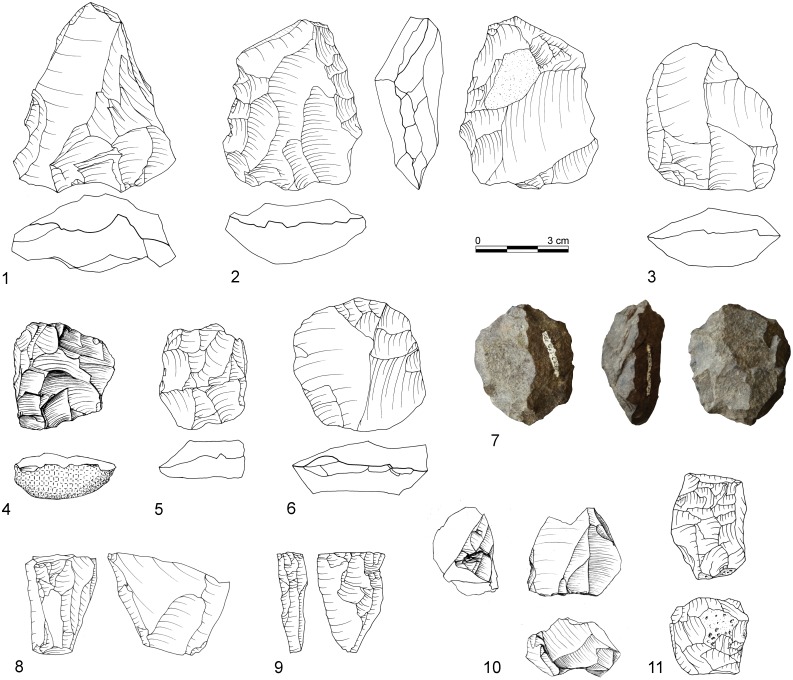
Core types from BM-BSP. 1: Parallel core (BSP, dolerite, E3-122); 2: Parallel core (BSP, hornfels, E3-206); 3: Parallel core (BSP, dolerite, C2-9); 4: Parallel core (BM, dolerite, D3-761); 5: Parallel core (SPCA, hornfels, E3, 550); 6: Parallel core (SPCA, dolerite, D2-243); 7: Inclined core (BSP, dolerite, C3-79); 8: Platform core, laminar products (SPCA, dolerite, E2-208); 9: Platform core, laminar products (SPCA, hornfels, C3-257); 10: Platform core (SPCA, dolerite, C3-149); 11: Platform core (BSP, hornfels, E2-16.1). Drawings 4 & 10 by F. Brodbeck; drawings 1–3, 5, 6, 8, 9, 11 by M. Malina; photograph 7 by M. Will. 4 & 10 after [63] Fig. 4.

**Figure 11:**
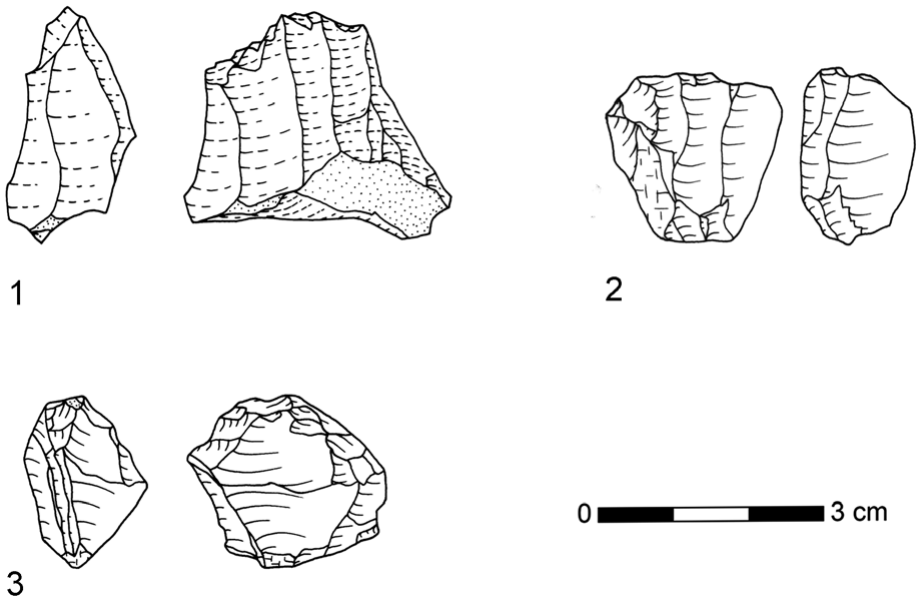
Selection of bladelet cores from BSP (1–2) and SPCA (3). 1: BSP, quartz, E3-273; 2: SPCA, hornfels, C3-149; 3: BSP, hornfels, D3-64.10. Drawings by M. Malina.

The most frequent core types are parallel (n = 23) and platform (n = 19) variants (Table 5). Among the remaining specimens there are three inclined, three bipolar, and four indeterminate broken cores. In total, the sample of cores is small for most assemblages. The uppermost layers BSP and SPCA show a strong dominance of parallel and platform cores, as does layer IV (Figure 10: 1–6; 8–11). All assemblages but MA feature parallel cores, many of which can be attributed to a Levallois system of reduction (*sensu* [98], [125], [126]). Inclined core variants, for the most part showing a discoid reduction method (*sensu* [125], [127]), occur exclusively in BSP and IV (Figure 10: 7). Only BSP features bipolar cores (n = 3). Most of the cores show traces from the production of flakes (n = 31), followed by blades (n = 14), bladelets (n = 5) and convergent flakes (n = 2). All bladelet cores are derived from the two uppermost layers BSP and SPCA (Figure 11). However, the majority of cores is heavily reduced and thus provides only limited information from the final stages of core reduction. In order to overcome these shortcomings and gain a better understanding of the core reduction systems in layers BM-BSP, we studied the geometry and configuration of dorsal negatives on debitage products and cores in more detail. Three coexisting strategies of core reduction characterize the assemblages: Parallel (mostly Levallois), platform, and inclined (discoid).

**Table 5:** Distribution of core categories^1^.

Layer	Parallel	Platform	Inclined	Bipolar	Indeterminatebroken
BSP	6	5	2	3	3
SPCA	8	4	-	-	-
CHE	2	1	-	-	-
MA	-	1	-	-	-
IV	5	7	1	-	1
BM	2	1	-	-	-
Total	23	19	3	3	4

1 Core classification follows the taxonomy of Conard *et al.* [104].

Parallel cores occur frequently. They are characterized by two hierarchical, asymmetric and non-interchangeable surfaces, sometimes with intense preparation of the striking platform (Figure 10: 1–6). The side of the core opposite to the removal surface is either steeply prepared or covered with cortex. Knappers prepared the lateral and distal edges of the core with centripetal removals to create a convex removal surface. Both end products and core rejuvenation flakes occur for this reduction strategy. The products of this system include (convergent) flakes which are longer than wide but also blades. Platforms of these products are often facetted. The (convergent) flakes are mostly flat, have feathered terminations, and exhibit exterior platform angles (EPA) that are typically >80°. The majority of the parallel cores, flakes and maintenance products demonstrates unidirectional recurrent (Figure 10: 1, 2) or centripetal removals (Figure 10: 3, 5, 6). Knappers also removed blades in a unidirectional and recurrent manner from the parallel cores. These products are mostly flat and frequently exhibit facetted striking platforms.

The second strategy of core reduction that we observed is a platform method aimed at the production of blades (Figure 10: 8, 9), flakes (Figure 10: 10, 11) and bladelets (Figure 11). Knappers often set up multiple striking platforms with several removal surfaces and rotated the core during reduction. They reduced the platform cores from both broad and narrow surfaces. The blades from these cores are characterized by plain striking platforms, an average width of ca. 19 mm and regular parallel edges. Most of the blades show recurrent unidirectional removals on the dorsal surface, but bidirectional patterns occur in lower numbers as well. From the six studied assemblages, only BSP (n = 4) and SPCA (n = 1) yielded cores for the production of bladelets (Figure 11). These cores demonstrate plain striking platform from which several bladelets are struck in a recurrent manner from one removal surface. The bladelet products are largely missing in BSP and SPCA.

A small number of cores and blanks also attests to the existence of an inclined reduction strategy with non-hierarchical and interchangeable surfaces without platform preparation, which appears to be confined to dolerite. Knappers reduced these cores by alternating removals from both surfaces around the entire circumference (Figure 10: 7). Products of this reduction sequence include the characteristic and frequent core edge flakes, in which the roughly triangular blank preserves part of the steep circumference of the discoid core on one lateral edge. The other main products of this method are short quadrangular flakes with inclined dorsal negatives and low EPAs (<80°).

In addition to these three main systems, we observed bipolar knapping on a few cores and flakes. This system of core reduction, however, occurs in very low frequencies and does not appear to be as structured and frequent as the other three methods. Furthermore, a total of 13 splintered pieces indicate a bipolar use of these specimens (*cf*. [128]).

#### Knapping technique

The inhabitants at Sibudu employed different knapping techniques depending on the blank type they produced. In all assemblages, flakes and convergent flakes were predominantly knapped using a hard stone hammer with direct and internal percussion. These products demonstrate an average platform thickness of around 6 mm in each assemblage (n = 1241) with very few butts thinner than 2 mm (4%). Bulbs are very frequent (72%) and often strongly developed with visible contact points or cones of percussion. Lips occur in low frequency (10%) and EPAs cluster around 85–90°. The high frequency of longitudinal breaks on flakes is also consistent with strong forces exerted by hard stone hammers that had direct contact with the core.

The knappers used a different approach to the production of laminar products. Based on approaches of previous studies [35], [129], we recorded a list of attributes and measurements on blades for each assemblage (Table 6). The analyzed sample amounts to 393 blades. The results show that bulbs are abundant (60%) but poorly developed. Proximal lips occur frequently (24%) and shattered bulbs constitute an even more common feature (31%). The blades feature prepared platforms (facetted 17%, dihedral 5%), but the majority of butts are plain (44%) or crushed (26%). Blade platforms are relatively thick with an average of 5.0 mm and a modal value of 3.0 mm. The EPAs cluster around 80°. We frequently observed contact points on the blades but almost no platform abrasion. Knappers often trimmed the proximal edges by small overhang removals prior to the production of a blade.

**Table 6:** List of attributes and measurements recorded on blades to diagnose the knapping technique.

**Discrete attributes**
- Presence of bulb of percussion (Y/N)
- Presence of proximal lip (Y/N)
- Presence of shattered bulb (Y/N)
- Presence of proximal trimming negatives (Y/N)
- Presence of abrasion on platform (Y/N)
- Presence of contact point of hammerstone (Y/N)
- Presence of (partial) Hertzian cone (Y/N)
- Type of platform (plain, facetted, dihedral, cortical, crushed)
**Measurements**
- Platform thickness (in mm)
- Platform width (in mm)
- Exterior platform angle (in degrees)

In summary, the discrete and metric attributes indicate that knappers predominantly used a soft stone hammer with direct internal percussion to produce blades. The abundance of shattered bulbs and contact points, the frequent occurrence of poorly developed bulbs and proximal lips, and the range of EPAs are consistent with results from experimental knapping with soft stone hammers [129], [130], although these experiments were performed on flint. A marginal percussion movement can be ruled out by the low frequency of platforms <2 mm (6%) and the lack of platform abrasion prior to blade removal. The fact that all four hammerstones found in BM-BSP are out of sandstone supports our findings.

#### Flaking efficiency and reduction intensity

**Figure 12:**
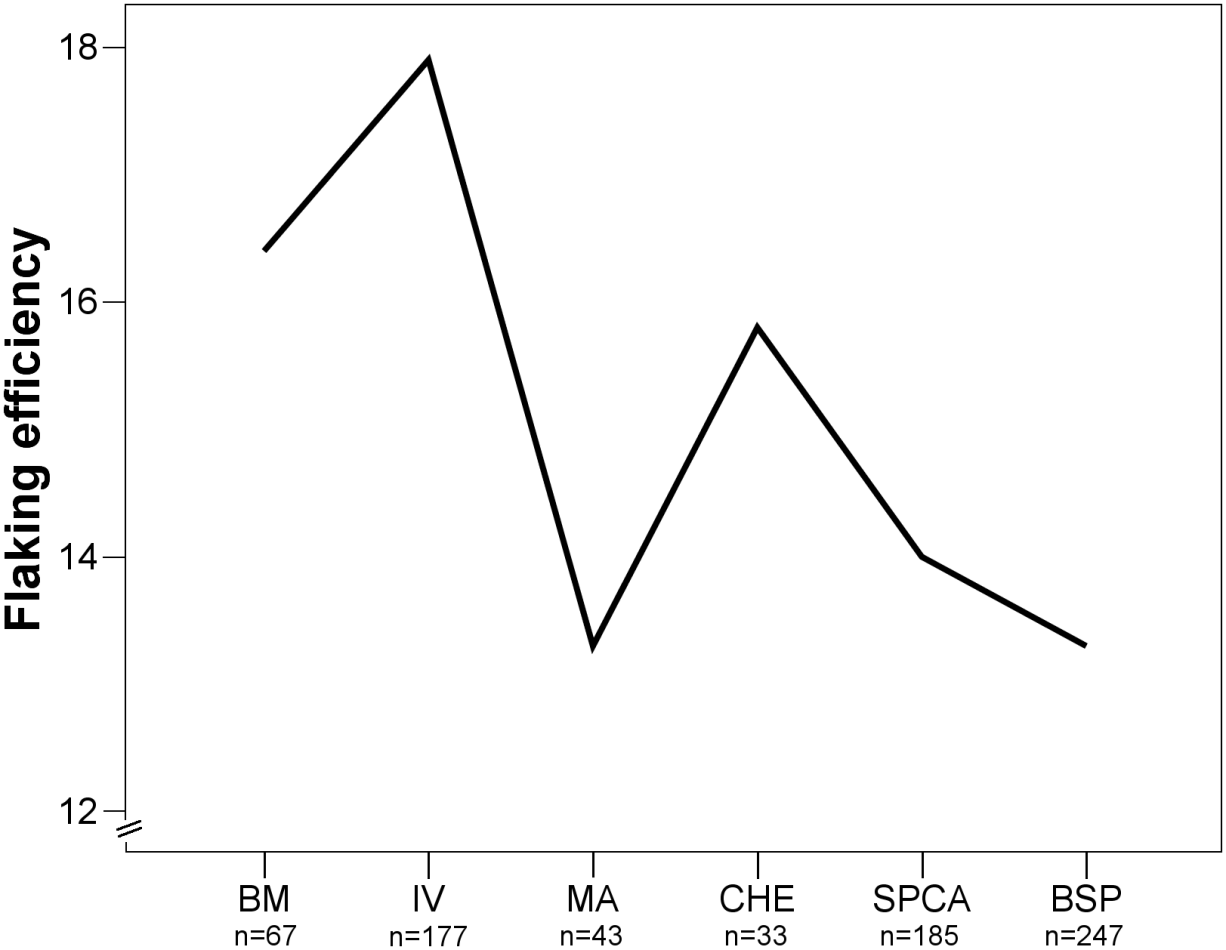
Mean values of flaking efficiency for BM-BSP. Flaking efficiency = edge length/mass. BM = oldest layer; BSP = youngest layer.

We found a strong temporal trend in the diachronic comparison of flaking efficiencies (Figure 12). The oldest layers BM and IV yield the highest values for flaking efficiencies. In contrast, the minimum values come from the youngest levels BSP and SPCA, suggesting that knappers made less efficient use of stone materials in these assemblages.

Concerning the reduction intensity of the assemblages, there is a clear separation between two groups for the ratios of blanks to cores. Highly reduced assemblages include BM and MA with values of 123∶1 and 66∶1. In contrast, BSP, SPCA, CHE and IV yield consistent blank to core ratios that are far lower (33–38∶1). Due to the low number of cores in some of the assemblages, these results need to be considered with caution. We thus also analyzed the sizes of flakes and cores, finding a consistent increase through time. The oldest assemblages BM and IV yield the smallest and thinnest blanks and cores, while the youngest assemblages (e.g. BSP, SPCA) demonstrate larger and thicker specimens. Blanks >80 mm occur only in the uppermost assemblages. Hence, the inhabitants at Sibudu reduced their lithic raw material more intensively in the earlier assemblages compared to the younger ones.

### Tool Assemblages

**Figure 13:**
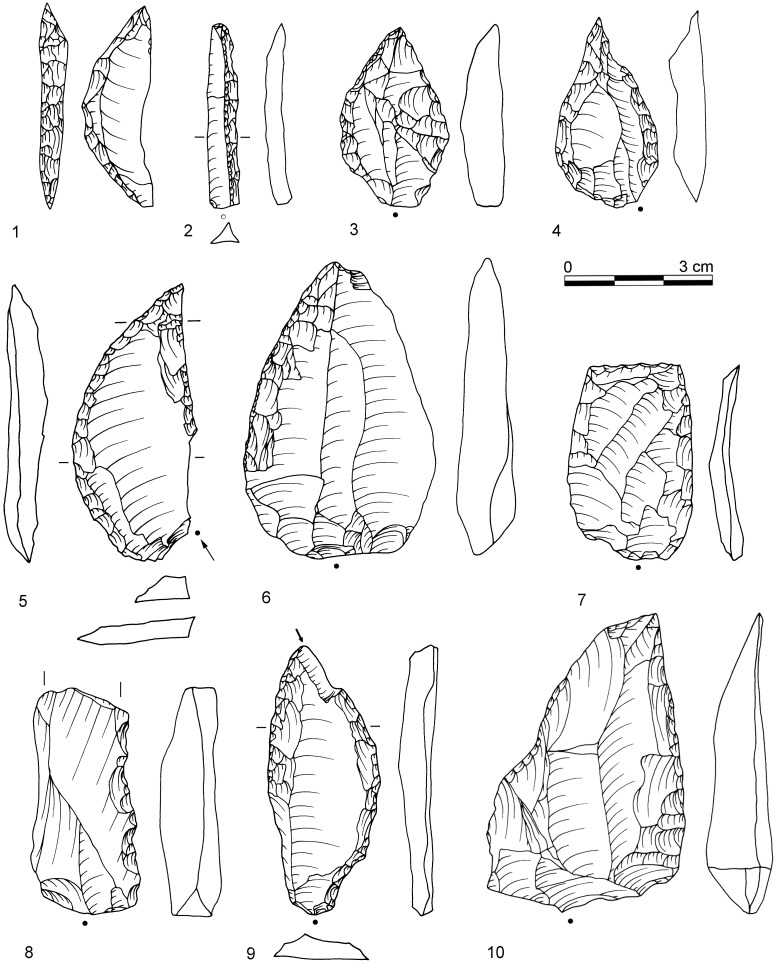
Selection of traditional tool types from BM-BSP. 1: Backed tool/segment (BSP, hornfels, D3-42.1); 2: Backed tool (BSP, hornfels, D3-17); 3: Unifacial point (BSP, hornfels, C3-42); 4: Unifacial point (BSP, hornfels, E3-40); 5: Unifacial point (BSP, hornfels, D3-18); 6: Unifacial point (BSP, hornfels, C2-8); 7: Biseau (IV, hornfels, E3-542); 8: Denticulate (IV, hornfels, D2-374); 9: burin (SPCA, hornfels, C3-273); 10: Side scraper (BSP, hornfels, C2-186). Drawings by M. Malina.

**Figure 14:**
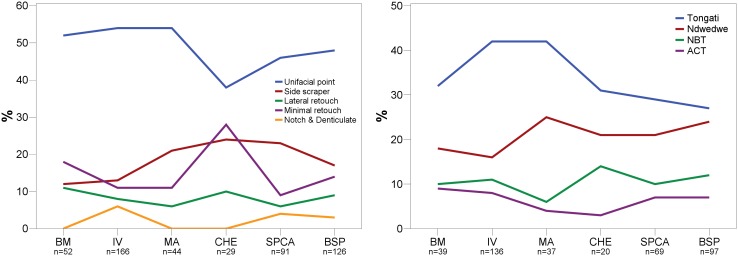
Percentual abundance of classic typological categories (left) and techno-functional tool classes (right). BM = oldest layer; BSP = youngest layer.

From a traditional typological point of view, unifacially retouched points characterize the six studied Sibudan assemblages (Figure 13). Unifacial points (*n* = 277) make up half of all modified pieces (*n* = 555) and constitute the most frequent tool type in each assemblage ranging between 38–54% (Figure 14; Table 7). They are followed by far fewer scrapers (17%) and lateral retouch on blades (8%). Other tool types that are usually frequent in MSA assemblages, like notches, denticulates, or splintered pieces, occur rarely (<3%). In some layers, these implements are absent (e.g. BM and CHE). Layers BM-BSP yield only 4 backed tools or segments (Figure 13: 1, 2) and 3 bifacial points. There is a marked increase of scrapers in the upper layers BSP-MA (17–24%) compared to the oldest assemblages IV (13%) and BM (12%). In general though, the range and frequency of tool types is homogenous.

**Table 7:** Distribution of traditional tool types.

Layer	Unifacial Point	Side Scraper	Lateral Retouch	Denticulate & Notch	End Scraper	Backed tool	Bifacial Point	Hammer-stone	Minimal retouch	Other
BSP	66 (48%)	24 (17%)	13 (9%)	4 (3%)	2 (1%)	2 (1%)	2 (1%)	1 (1%)	19 (14%)	6 (4%)
SPCA	48 (46%)	24 (23%)	6 (6%)	4 (4%)	3 (3%)	-	-	2 (1%)	9 (9%)	8 (8%)
CHE	11 (38%)	7 (24%)	3 (10%)	-	-	-	-	-	8 (28%)	-
MA	26 (54%)	10 (21%)	3 (6%)	-	1 (2%)	-	-	-	5 (11%)	3 (6%)
IV	97 (54%)	23 (13%)	15 (8%)	11 (6%)	1 (1%)	2 (1%)	1 (1%)	1 (1%)	20 (11%)	8 (4%)
BM	29 (52%)	7 (12%)	6 (11%)	-	1 (2%)	-	-	-	10 (18%)	3 (5%)
Total	277 (50%)	95 (17%)	46 (8%)	19 (3%)	8 (1%)	4 (1%)	3 (1%)	4 (1%)	71 (13%)	28 (5%)

Rounded percentages are given in brackets.

From a techno-functional point of view, four formal tool classes and tool cycles characterize BM-BSP (see Figures 3–5): Tongatis, Ndwedwes, naturally backed tools (NBT), and asymmetric convergent tools (ACT). The four formal tool classes make up more than two thirds in each assemblage (67–77%; Table 8). Throughout the sequence, Tongatis are the most abundant tool class (27–42%), followed by Ndwedwes (16–25%). Tongatis and Ndwedwes thus constitute the hallmark of formal tools in BM-BSP, representing >50% of each assemblage with a combined total of 301 pieces (Figure 14). NBTs (Figure 5: 1–6) and ACTs (Figure 5: 7–10) occur in low but stable frequencies throughout the sequence (NBTs: 6–14%; ACTs: 3–9%). Other formal tools, comprising various forms of scrapers, denticulates and notches, play a minor role (3–13%).

**Table 8:** Distribution of techno-functional tool classes.

Layer	Tongati	Ndwedwe	NBT	ACT	Biseau	Splintered piece	Formal tool	Brokentool	Other
BSP	37 (27%)	34 (24%)	17 (12%)	9 (7%)	2 (1%)	4 (3%)	18 (13%)	15 (11%)	3 (2%)
SPCA	30 (29%)	22 (21%)	10 (10%)	7 (7%)	-	4 (4%)	13 (12%)	16 (15%)	2 (2%)
CHE	9 (31%)	6 (21%)	4 (14%)	1 (3%)	-	-	2 (7%)	7 (24%)	-
MA	20 (42%)	12 (25%)	3 (6%)	2 (4%)	-	1 (2%)	4 (8%)	6 (13%)	-
IV	74 (42%)	29 (16%)	19 (11%)	14 (8%)	2 (1%)	3 (2%)	13 (7%)	22 (12%)	2 (1%)
BM	18 (32%)	10 (18%)	6 (10%)	5 (9%)	-	1 (2%)	2 (3%)	13 (23%)	2 (3%)
Total	188 (34%)	113 (20%)	59 (11%)	38 (7%)	4 (1%)	13 (2%)	52 (9%)	79 (14%)	9 (2%)

Rounded percentages are given in brackets.

We also examined technological aspects to assess the approach of knappers to execute retouch. The inhabitants preferentially selected elongated (18.5%) and convergent forms (33.5%) for secondary modification (Table 9). Still, most tools are made on regular flakes (48%). The knappers applied retouch predominantly to the dorsal side of the blanks (93%) and only in rare instances on the ventral side (3%) or bifacially (4%). Small stepped negatives are the most abundant type of modification on tool edges. Many times the retouch on tools is intense and invasive, with several layers of small overlapping negatives. The modification often covers long parts of the artifact edges, indicating abundant retouch and recycling activities taking place on-site. Concerning the preservation of tools, only a third is in complete state.

**Table 9:** Number of blank types used for the manufacture of tools for the combined assemblages BM-BSP.

Blank	Tools (n)	Tools (%)^1^	Blanks (%)^2^	%diff^3^
Flake	262	47.5	71.0	−23.5
Convergent Flake	184	33.5	13.1	+20.4
Blade	102	18.5	15.5	+3.0
Bladelet	3	0.5	0.4	+0.1

1 Proportion of tools made on this blank type in all assemblages.

2 Proportion of blanks in all assemblages.

3 Tools (%) – Blanks (%).

### Reduction Sequences

We characterized reduction sequences for the different raw materials within each assemblage. In general, both the local dolerite and the non-local hornfels show complete reduction sequences, with products of all manufacturing phases present, indicating their on-site production. Having said this, hornfels exhibits a strong emphasis on the production, resharpening and curation of tools. In contrast to dolerite and hornfels, quartzite, jasper and CCS typically occur in the form of isolated blanks and tools. Sandstone and quartz are only represented by the early stages of knapping. For the latter, there are many cores (n = 3) compared to the few debitage products, suggesting that people exported quartz artifacts to other places in the landscape.

We analyzed the reduction sequences in more detail for the largest assemblage BSP including all artifacts >25 mm (n = 822). BSP mirrors the general observations previously discussed very closely. Dolerite exhibits all stages of the knapping process in relatively large numbers (84% blanks, 2% cores, 11% tools) with some highly cortical products from the earliest phases of reduction. Hornfels displays an emphasis on the distal reduction phases (66% blanks; 29% tools) and an underrepresentation of early manufacturing stages. Quartzite, sandstone and CCS appear in very low numbers and exclusively as finished blanks and tools. The early stages of production for these raw materials presumably occurred off-site during their procurement and previous use. The five quartz artifacts from BSP include three cores but only two unmodified flakes, demonstrating an apparent lack of debitage products. The existence of a quartz bladelet core (Fig. 10: 1) and the absence of the corresponding bladelets in BSP support the observation that the inhabitants of Sibudu transported quartz artifacts outside the area of excavation.

Quantitative data support the qualitative observations of reduction stages taking place at Sibudu. The proportion of cortex on an artifact, whether from an outcrop or pebble source, can inform on its position in a reduction sequence as cortex cover decreases in a more or less continuous manner during the knapping process [93], [131], [132]. We assessed cortex on each artifact in increments of 20% from completely non-cortical (0%) to fully cortical (100%) and compared the results between layers and raw materials. In general, all Sibudan assemblages show a similar pattern in which all classes of cortex cover occur (Table 10). Non-cortical specimens amount to ∼60–65%. The number of artifacts per increment class decreases gradually with higher cortex proportions. While there are many cortical specimens (>50%), fully cortical artifacts are rare (0–2%), suggesting that the initial stages of decortification took place off-site. There are some assemblages with more cortical pieces (e.g. CHE, MA) than others (e.g. BSP, BM), but there is no consistent diachronic trend.

**Table 10:** Cortex cover on artifacts for each assemblage and for the total sample of dolerite and hornfels.

	**Layer**						**Raw material**	
**% cortex**	**BSP**	**SPCA**	**CHE**	**MA**	**IV**	**BM**	**Dolerite**	**Hornfels**
0	525 (64%)	368 (64%)	74 (56%)	109 (61%)	410 (61%)	174 (66%)	963 (58%)	626 (70%)
1–20	124 (15%)	73 (13%)	20 (15%)	20 (11%)	94 (14%)	30 (11%)	232 (14%)	115 (13%)
21–40	88 (11%)	65 (11%)	18 (14%)	22 (12%)	75 (11%)	26 (10%)	200 (12%)	81 (9%)
41–60	31 (4%)	23 (4%)	8 (6%)	10 (6%)	45 (7%)	16 (6%)	92 (6%)	34 (4%)
61–80	34 (4%)	35 (6%)	6 (4%)	12 (7%)	36 (5%)	10 (4%)	99 (6%)	30 (3%)
81–99	15 (2%)	8 (1%)	5 (4%)	2 (1%)	14 (2%)	4 (2%)	42 (3%)	4 (1%)
100	5 (0%)	6 (1%)	2 (1%)	3 (2%)	2 (0%)	2 (1%)	17 (1%)	3 (0%)
Total	822	578	133	178	676	262	1645	893

Rounded percentages are given in brackets.

We also compared the cortex cover of artifacts made from dolerite and hornfels (Table 10). In general, both dolerite and hornfels show all proportions of cortex in each assemblage, indicating complete reduction sequences that took place on-site. For hornfels, however, there are more non-cortical specimens whereas dolerite exhibits more highly cortical artifacts (>50%). Only BSP and SPCA yielded enough quartzite specimens to roughly assess its cortex frequencies. In BSP and SPCA combined, only 1 out of 19 specimens show any amount of (pebble) cortex, indicating that knappers reduced quartzite mostly off-site.

In order to study the retouch and curation activities of the inhabitants, we quantified the retouch debitage among the small debitage for each raw material (<25 mm; see [63], [133]). We analyzed a sample of small debitage from each assemblage (total *n* = 8193). On average, retouch flakes amount to ∼16% ([63], Tab. 3). The percentages fluctuate between 10–25%, suggesting extensive retouch and curation activities performed on-site throughout the sequence. This observation corresponds to the very high proportion of tools in these layers compared to many other MSA assemblages. The characteristics of the retouch flakes such as very low EPAs, the presence of a lip and diffuse bulbs of percussion attest to soft hammer percussion with a tangential knapping motion.

**Figure 15:**
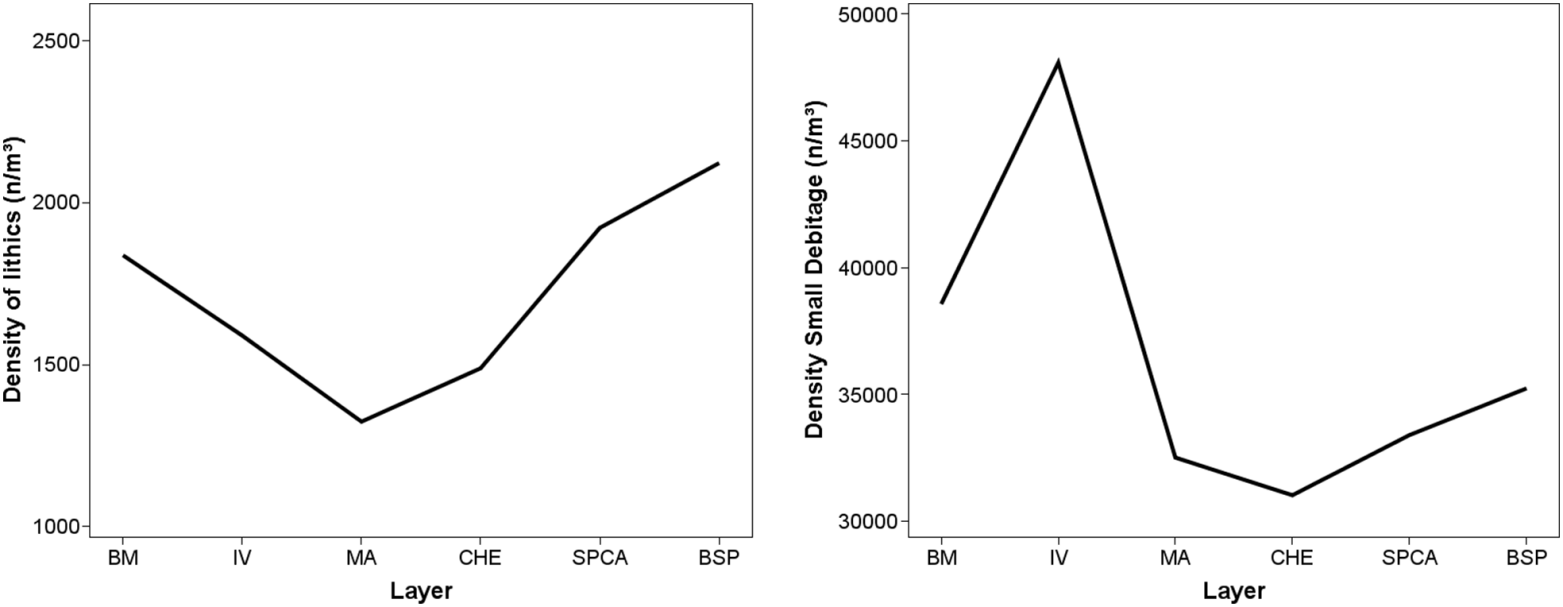
Density of lithic remains throughout BM-BSP. Lithics >25 mm (n/m^3^, left) and lithic remains <25 mm (n/m^3^, right). BM = oldest layer; BSP = youngest layer.

The density of lithic artifacts (>25 mm) and small debitage (<25 mm) can help to assess the intensity of on-site reduction and site use. Figure 15 illustrates the densities of stone artifacts in layers BM-BSP, ranging between 30,000–50,000 n/m^3^ for lithic products <25 mm. Compared to values of South African MSA sites like Pinnacle Point 13BB (<5000 n/m^3^ for all occupation horizons; [134]) and our own excavations at Hoedjiespunt 1 ([135], ∼600–3000 n/m^3^, unpublished data) the small debitage values are very high, suggesting repeated and intense occupations with widespread knapping activities taking place. There are, however, strong temporal fluctuations in the lithic densities, suggesting differing intensities of on-site stone knapping. The higher small debitage densities in BM and especially IV are roughly consistent with the observations that these assemblages are more intensively reduced.

### Raw Material Economy

The knappers at Sibudu used their main raw materials in a different manner. Observations from the reduction sequences demonstrated that the non-local hornfels shows an emphasis on the production and curation of tools. The Raw Material Retouch Index (RMRI; [136]) supports this interpretation. Blanks made from hornfels (RMRI = 1.43) were more likely to be retouched than dolerite (RMRI = 0.81). The results from the debitage analyses by raw materials are also consistent with these observations. We found an overrepresentation of hornfels tools (48%) compared to the overall proportion of this raw material in the entire assemblage (34%). The ratio of tools to blanks is on average two times higher for hornfels compared to dolerite. In contrast, dolerite occurs most often in the form of unmodified blanks, with a marked underrepresentation of retouched pieces.

An independent t-test comparison of the weight, maximum dimensions and thickness of all complete tools (Table 11) shows that retouched artifacts from hornfels are significantly lighter, smaller and thinner than those from dolerite (*p*<0.002). Principally the same statistical results are obtained for the differences in maximum core dimension, weight and thickness between the two raw materials, with dolerite cores being significantly heavier, larger and thicker (*p*<0.031). Hornfels also exhibits by far the smallest, lightest and thinnest blanks of all raw materials. The difference to unretouched dolerite blanks is highly significant (*p*<0.001).

**Table 11:** Independent t-test comparison of metric attributes between complete artifacts made from dolerite and hornfels.

	Ø MD (mm)^1^	Ø Thickness (mm)	Ø Weight (g)
**Tools**			
Dolerite	46.4	9.6	14.4
Hornfels	42.5	8.1	7.4
*df* 2	430	430	430
*p* 3	0.002	<0.001	<0.001
**Cores**			
Dolerite	54.8	22.6	66.8
Hornfels	44.6	17.7	29.1
*df*	39	39	39
*p*	0.031	0.028	0.005
**Blanks**			
Dolerite	45.0	8.8	13.4
Hornfels	40.2	6.7	6.9
*df*	1459	1459	1459
*p*	<0.001	<0.001	<0.001

1 Maximum dimension of the artifact.

2 Degrees of freedom.

3 Significance value of the two-sided t-test (α = 0.05).

The knappers also varied their approach to core preparation with regards to raw materials as can be deduced from the types of platforms. Hornfels has the highest proportion of prepared platforms (29%), followed by dolerite (24%), and sandstone (19%). Very fine platform preparation with >5 small facets occurs most often on hornfels artifacts. In correspondence with this pattern, plain butts are far more frequent for dolerite than hornfels. In contrast, platform crushing and shattering is mostly associated with hornfels and quartzite, probably due to their more delicate nature. Regarding blank types, knappers produced flakes predominantly from dolerite, quartzite and sandstone. Quartz, jasper and CCS occur only in the form of flakes. The relative frequency of blades and convergent flakes is highest for hornfels, with dolerite being second. For hornfels, there are some very long blades and elongated convergent flakes with intense proximal overhang removals and abundant facettation of platforms. Some tool types also show a favored use of raw materials. Knappers made splintered pieces predominantly from hornfels while dolerite was preferentially used to manufacture notches and denticulates. In terms of techno-functional tool classes, knappers at Sibudu preferred hornfels for producing Ndwedwes and dolerite for the manufacture of NBTs.

**Figure 16:**
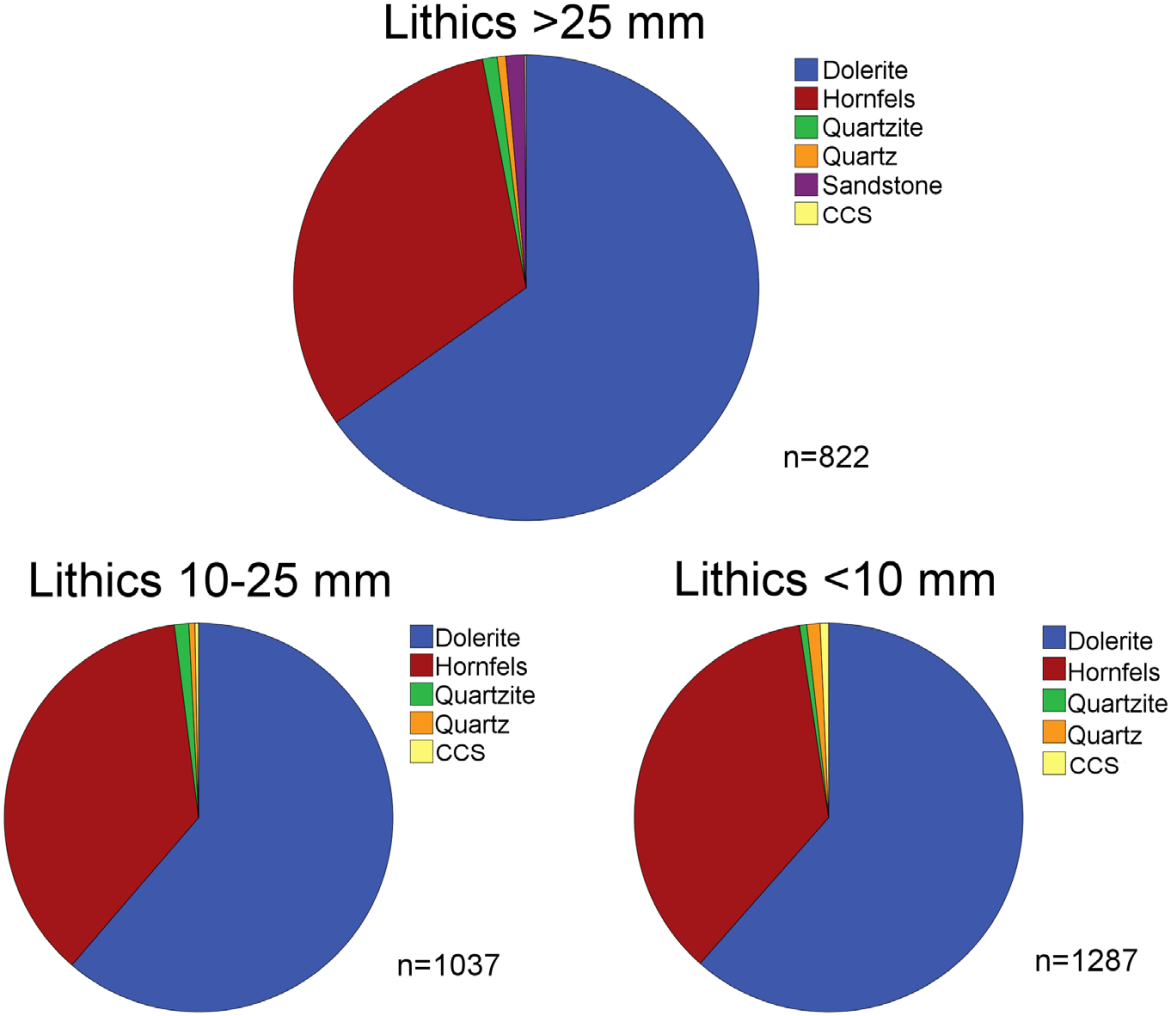
Proportions of raw materials in assemblage BSP. Lithics >25 mm (top), lithics 10–25 mm (bottom left) and lithics <10 mm (bottom right).

The amount of small debitage products can provide information on the reduction of raw materials on-site [137]–[139]. We quantified a sample of small debitage products by raw materials in BSP (*n* = 2324). The resulting frequencies for hornfels and dolerite compare well with the abundances of artifacts >25 mm (Figure 16), demonstrating that knappers reduced both materials on-site. Consistent with their incomplete reduction sequences, small debitage products of quartzite, other raw materials and especially quartz are rare. Preliminary observations on the very large assemblage of small debitage products from the other layers (*n* = 43097) are consistent with these results. In each layer, there is abundant small debitage for dolerite and to a lesser degree for hornfels. In contrast, small knapping products for quartzite, quartz and other raw materials occur rarely. In terms of flaking efficiency, hornfels demonstrates the highest value among all raw materials, followed by dolerite with markedly lower values (Table 12). Sandstone and quartzite show the lowest edge length to mass ratios. These results suggest that among all raw materials, knappers used hornfels in the most efficient way, presumably to conserve this high-quality and non-local raw material.

**Table 12:** Flaking efficiency by raw material for the combined assemblages BM-BSP.

Raw material	n	Flakingefficiency Ø^1^	Max.	Min.	SD
Dolerite	734	14.3	60	2	9.2
Hornfels	283	19.6	89.9	3.9	11.1
Quartzite	10	11.9	30.9	3.3	9.5
Sandstone	11	12.7	30.2	1.9	8

1 Flaking efficiency is calculated after [120].

The observed patterns of raw material economy occur alike throughout BM-BSP. Analyses of reduction sequences, frequencies of retouched forms, RMRI values, small debitage products, and flaking efficiencies suggest a stronger emphasis on retouch and curation for hornfels, with knappers investing more energy and time in the treatment of this non-local high-quality raw material compared to dolerite. An additional factor probably influenced these differences. While hornfels is fine-grained and easy to knap, its sharp edges are often fragile and have a tendency to break. Thus, they require more resharpening than the more durable tool edges of the coarser-grained dolerite (see [123]).

## Discussion

### Key Elements and Technological Variability of the Sibudan Lithic Assemblages BM-BSP

The period of the MSA following the HP in southern Africa (“post-HP”) has not been studied in great detail, particularly in comparison with the HP and SB technocomplexes (see [19], [35], [39], [52], [54], [60], [63], [64]). We examined six lithic assemblages from Sibudu that post-date the HP, from the so-called Sibudan (*sensu* [63]), as part of the process of correcting this research bias.

The lithic assemblages of the Late Pleistocene sequence at Sibudu that we have analyzed here yield a robust technological signal. The key elements of BM-BSP include technological, techno-economic, techno-functional and typological aspects. These characteristics occur in a homogenous manner in each assemblage and can thus help to define features of the Sibudan (*sensu* [63]). The lithic assemblages demonstrate that the inhabitants followed a consistent pattern of raw material procurement in the brief period we have studied so far, both in terms of their variety and abundance. Knappers used tool stones of local (dolerite, sandstone, quartzite) and non-local (e.g. hornfels) origin. We also observed a uniform approach to the use of the two main raw materials dolerite and hornfels in terms of reduction sequences and the production of blanks. In accordance with its transport distance and high quality, people curated artifacts of hornfels more intensively than those of dolerite (*cf.* [121], [122]). Our results are in agreement with observations from other research [58], [123] suggesting that knappers had ready access to dolerite.

All the Sibudan assemblages we have studied so far are based on various blank types of large size (40–48 mm on average). Throughout BM-BSP, knappers produced blades with principally the same dimensions and shapes. Elongated and convergent products were preferentially selected for retouch and exhibit higher frequencies of prepared platforms. Furthermore, the co-existence of several reduction methods characterizes the layers of this study. Parallel and platform systems are frequent, with inclined cores playing a minor role. Only the parallel cores show extensive core preparation, with one quarter of all blanks exhibiting facetted platforms.

Knappers typically employed hard stone hammers to produce (convergent) flakes but soft stone hammers for blades in all assemblages. The proportion of retouched artifacts is exceptionally high among pieces >25 mm (17–27%), with a diverse and distinct inventory of formal tools. From a traditional point of view, unifacial points constitute the hallmark of implements in BM-BSP, while other typical MSA tools like denticulates and notches occur rarely. From a techno-functional perspective, four tool classes which amount to more than two thirds of all retouched specimens characterize the assemblages. The large number of Tongatis, Ndwedwes, NBTs and ACTs is a characteristic feature of the assemblages BM-BSP. The highly repetitive pattern of organizing the working edges for these implements also indicates a structured approach to tool manufacture, providing distinctive and well-defined tool cycles (see also [63]). We do not consider these tool classes as type fossils but as organizational elements within the Sibudan. They also occur in other periods at Sibudu, and their abundance will likely vary in other parts of the sequence pre- and post-dating the HP. We are currently working to refine this approach using a longer sequence of the Sibudan.

Finally, the six Sibudan assemblages document that similar knapping activities have been performed at the site. Throughout this part of the sequence, we found that the same stages of reduction taking place for each raw material. While dolerite and hornfels show mostly complete reduction sequences, quartzite, quartz, sandstone, jasper and CCS exhibit truncated manufacture sequences. The most prominent feature of the assemblages BM-BSP is their strong emphasis on the distal part of the reduction sequence. Compared to many other assemblages from the MSA, these layers exhibit a very high abundance of tools with intensively retouched and curated pieces as well as a large amount of retouching debitage. This observation is related to the intensive production and curation of tools in these layers. Of course, it is possible that other facies of the Sibudan show different features including lower proportions of tools and distal elements in the lithic technology.

While the Sibudan assemblages studied so far provide a strong and consistent technological signal, the high-resolution stratigraphy allowed for the recognition and evaluation of small-scale technological variation throughout the archaeological deposits. This behavioral variability is to be expected since the technological behavior of mobile hunter-gatherer groups is influenced by many ecological, social and functional parameters that change within short periods of time at the same locality (e.g. [140]–[142]).

**Figure 17:**
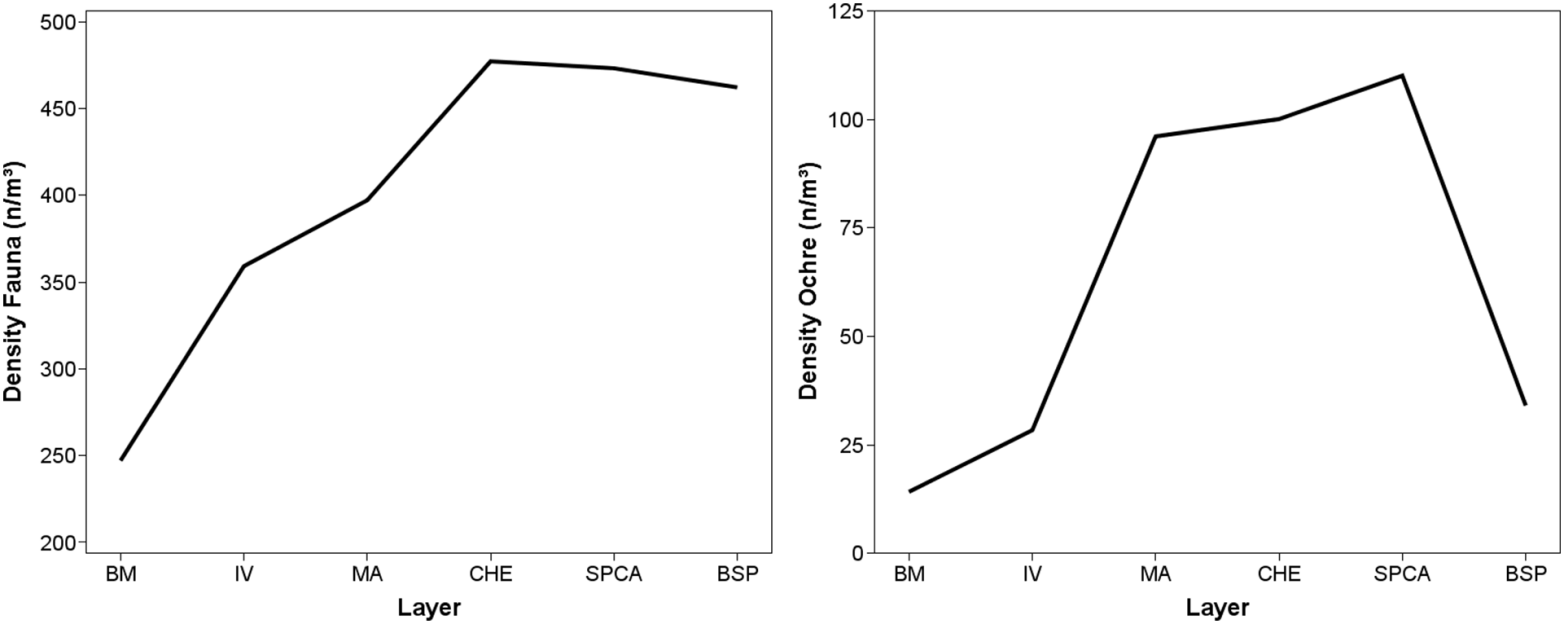
Density of faunal remains and ochre throughout BM-BSP. Faunal remains >25 mm (n/m^3^, left) and ochre >25 mm (n/m^3^, right). BM = oldest layer; BSP = youngest layer.

We observed slight differences in the choice of raw materials. While the main types of tool stones remain the same, rare variants such as CCS and jasper occur only in a few assemblages. The abundance of non-local raw materials ranges between 25–38%. These variations might reflect differential access to the sources of raw materials or changes in the mobility system of the inhabitants such as smaller or larger home ranges and foraging trips. There is also some variation in the forms of tools produced, although there are no clear temporal trends in this part of the sequence. This variability could be an outcome of different activities performed at the site. Future studies will investigate site function and tool use in more detail. Finally, the difference in the reduction intensities of the assemblages constitutes the most conspicuous technological variation in the studied sequence. The older assemblages (BM, IV) are more intensively reduced, with higher blank to core ratios and smaller debitage products. Consistent with this observation, these layers also feature the highest densities of small debitage. Interestingly, the density for ochre and faunal remains >25 mm that people left behind at the site does not follow the patterns of the lithics (Figure 17). These subtle distinctions between the upper and lower assemblages are most likely the result of differences in the use of the site, the access to raw materials and the system of mobility.

In contrast to studies which consider the “post-HP” as a phase of unstructured or unsophisticated lithic technologies during the MSA (e.g. [41], [50], [65], [67]), we found clear cultural signals that unite the assemblages studied at Sibudu so far. These key elements occur homogeneously in many independent aspects of the lithic technology in six successively stratified assemblages of different sample sizes and reduction intensities, attesting to a structured lithic technology. Many of these characteristics, such as the well-recognizable tool assemblages with repetitive forms and distinctive reduction cycles, or the production of morphometrically standardized blades by soft stone hammers, demonstrate that the people at Sibudu did not possess a rudimentary or unsophisticated approach to stone knapping (*contra* [41], [50], see also [63]).

### Comparing the Sibudan to MSA Assemblages following the HP in Southern Africa

In order to move forward with the process of characterizing the Sibudan, it is essential to compare its lithic assemblages with those from other sites of this time period. Only then will it be possible to assess the spatial and temporal variation of the material culture following the HP and to consider where the Sibudan fits in the African taxonomy with its hierarchy of phases defined at the Burg Wartenstein meeting of 1965 ([143], [144], see also [9]).

Recently, Lombard *et al.* [9] proposed the “Sibudu Industry” or “Sibudu technocomplex” to describe lithic assemblages at Sibudu that derive from both the “post-HP” (∼58 ka) and “late MSA” (∼48 ka) layers. They [9] view the Sibudu technocomplex as a pan-southern African phenomenon including assemblages from a list of ten sites that are characterized by the following typo/technological characteristics: most formal retouched is aimed at producing unifacial points which are predominantly produced by Levallois methods, with a tendency towards elongated forms with facetted platforms (*Sibudu point* as type fossil). Some plain butts occur as well. Side scrapers are present and there are rare bifacially retouched points and backed pieces [9], [145]. While our results from the lithic assemblages BM-BSP are broadly consistent with these characteristics, many important technological elements that we have found do not feature in this list. Detailed information on the methods of core reduction, the types of blanks produced, the knapping techniques and the reduction sequences will need to be provided for a conclusive comparison.

The most straightforward approach to evaluate the place of the studied Sibudan assemblages within the cultural sequence of the Late Pleistocene MSA are site by site comparisons. We chose assemblages based on the availability of technological data, reliable stratigraphy and secure dating. We also selected localities that are broadly comparable in their age, geographical and environmental parameters, and patterns of site-use, although this was not always possible. Lithic assemblages from the eastern part of South Africa constitute the most promising comparisons due to the short geographical distances and similar environmental circumstances. The southern African summer rainfall zone has provided several MSA sites that follow the HP (see [9], [54], [71]).

Umhlatuzana Rock Shelter (URS) lies in KwaZulu-Natal only 90 km south-west from Sibudu and ∼35 km from the Indian Ocean [73], [145]. The earliest layers that follow the HP (“late MSA”, Levels 19–21) date to around 40–44 ka [74]). While there are some problems with the stratigraphy [73], [145], recent OSL dating supports the integrity of the sediments [74]. In the following we describe the “late MSA” assemblages from URS (after [73], [145]) and also include detailed descriptions of the unifacial points [64], [74].

The lithic assemblages are large (17.000–70.000 pieces), suggesting intensive occupations and on-site knapping. Hornfels dominates the assemblages (60–90%), followed by quartzite (11–35%) and few other raw materials. Flakes with plain platforms constitute the most frequent blank type, but facetted butts occur as well. Bladelets are more frequent than blades, with the latter being rare (n = 36). Knappers manufactured bladelets from both platform and bipolar cores, with an average width of 6 mm. The most frequent core forms are irregular and platform types, with bipolar cores being less abundant. Kaplan [73] mentions prepared core technology but provides no further descriptions. The majority of cores is very small with mean lengths of ∼20 mm. Formal tools account for only 0.2%, but no size cut-off point was used for artifact counts [73], [145]. Unifacial points (37–40%) dominate the tool assemblages, followed by bifacial points (4–11%) and scrapers (3–15%). Rare miscellaneous backed pieces, backed points and small segments complete the tool spectrum. Knappers preferentially selected hornfels and elongated flakes to manufacture unifacial points [64], [74]. The points are generally of large size (∼48 mm) and feature facetted platforms (22%). URS displays a variety of unifacial point forms, often with invasive retouch. The majority of the depicted unifacial points resembles Tongatis (see in [64], Fig. 5a: c, f, g, i, l), but there are also three potentials Ndwedwes (see in [64], Fig. 5a: e, h) and one ACT (see in [64]; Fig. 5a: k).

Overall, the “late MSA” at URS conforms to the Sibudan assemblages BM-BSP in several typo-technological aspects. The core reduction methods are broadly similar and the unifacial points at URS match the variety in forms, the size, the intensive retouch and the blank types of those manufactured at Sibudu (*cf*. [64], [146]). Having said this, there are also differences. In contrast to the assemblages we have studied, URS features finely made bifacial points and very small backed segments. Additionally, the absolute number of retouched pieces (n = 217) in relation to the total assemblage (n = 130,000) is around five times lower for URS compared to Sibudu (tools n = 555; total assemblage n = 60,000). Discoid technology has not been reported at URS and it is unclear whether knappers produced convergent flakes. The abundance and small size of bladelets as well as the scarcity of blades also distinguishes URS. There are no information on rock type availability, raw material economy or knapping technique.

Rose Cottage Cave constitutes one of the few well-excavated, well-stratified and well-dated sites of eastern part of southern Africa [35], [147], [148]. The large cave lies in the Orange Free State ca. 350 km west of Sibudu. The early “post-HP” assemblages (THO, BYR) are dated to around ∼50 ka by TL or ∼57 ka by OSL [35]. We summarize the recent description of the lithic assemblages [35] with additional information from Harper [148].

The knappers at RCC used mostly local rocks, with more than 80% being opaline of high knapping quality, 10% tuff and few other raw materials. The inhabitants frequently produced blades (BYR 57%, THO 30%) but flakes are reported to be the primary objective of core reduction. The blades are mostly irregular, showing a low degree of standardization. Knappers produced blades by unidirectional reduction from the narrow face of the core. Cores make up 9–13% of the assemblages, with frequent bipolar cores in THO (n = 25) but not in BYR (n = 1). Flake cores dominate and Levallois flakes are common. The inhabitants used hard stone hammers with internal percussion to produce blades, often with facetted platforms (25%). Tool frequencies are high for both BYR (14.6%) and THO (26.5%). Various scraper forms dominate the tool assemblages (55%), followed by unifacial and partly bifacial points (12%), some scaled pieces and rare backed pieces, notches and denticulates. The tool types show little standardization. Flakes form 55–72% of blanks used for retouched pieces, with blades amounting to 28–45%. Unifacial points were predominantly made on flakes. Knappers manufactured most of their tools on opaline, corresponding to its overall abundance. The large number of small debitage pieces indicates frequent on-site tool manufacture.

There are several parallels to the Sibudan assemblages BM-BSP, including the production of both blades and flakes, Levallois and platform reduction methods, the high number of retouched specimens, the variety of tool forms and the manufacture of tools on-site. The abundance of fine-grained raw materials around the site explains the lack of non-local raw materials. In contrast to RCC, however, knappers at Sibudu produced blades from both narrow and broad surfaces of cores with higher degrees of morphometric standardization. They also employed a soft stone hammer for the production of blades. There is no information on the existence and role of convergent flakes as desired blanks at RCC. In opposition to Sibudu, cores are frequent in the early “post-HP” at RCC but without discoid reduction. The relatively low frequency of unifacial points at RCC might be partially explained by the separation of convergent scrapers and unifacial points [35]. Of the three depicted unifacial points, two compare well to Tongatis (see in [35], Fig. 16: 7–8) but none to Ndwedwes or asymmetric points. This observation matches with Harpeŕs [148] description that most unifacial points are thin and show symmetric triangular distal ends.

In a next step, we compared the six Sibudan lithic assemblages with geographically more distant areas of South Africa. Both the Southern (e.g. [10], [11], [149]) and Western Cape (e.g. [56], [59], [62], [150]) have provided several localities with lithic assemblages post-dating the HP. Klasies River (KR) is a complex of caves and shelters located on the southern coast of South Africa about 200 km east of Mossel Bay. The locality is famous for its almost 20 m thick sequence which long served as the type site for the cultural stratigraphy of the South African MSA [10], [11], [151]. Most recently, Wurz [11] and Villa *et al.* [60] studied the “MSA III” lithic assemblages of Cave 1A that date to around ∼58–60 ka [40], [152].

The majority of raw materials is local, including quartzite, quartz, hornfels and chalcedony. Silcrete constitute the only potential non-local tool stone and occurs in low frequencies (but see [153]). Knappers primarily manufactured blades (>50%), with convergent flakes being rare. According to Wurz [11] there are also no cores for convergent flakes. The main core reduction method is unidirectional blade removals from semi-prismatic cores, beginning on the narrow face of the core and using symmetrical crested blades (see in [60]; Fig. 16). Blade widths range widely between 10–30 mm and do not show a normal distribution around one peak (in [60]; S. Fig. 21). Knappers employed direct internal percussion with a hard stone hammer to produce blades. About 10% of the artifacts are retouched. Side scrapers, denticulates and notches dominate the tool assemblages, but truncated facetted pieces occur as well. Unifacial points are rare (7%; [60]), but Singer and Wymer [10] report ∼24%. Knappers preferentially selected blades (85%) over flakes (15%) for retouch. Almost all of the modified pieces are from the local quartzite, with few specimens from the potentially non-local silcrete.

Overall, the “MSA III” lithic assemblages at KR differ markedly from the Sibudan assemblages we have studied so far. While the existence of a blade production strategy with a comparable method of core reduction unites the assemblages, there are several major technological and typological differences. In BM-BSP flakes and not blades are the principal types of blanks produced, and discoid and Levallois core reduction method occur as well. The blades at Sibudu show higher standardization in size and shape, with a width distribution around a single peak. Furthermore, knappers usually manufactured blades with soft stone hammers and not hard stone hammers. While retouched specimens are relatively frequent at KR, the tool assemblages appear to be distinct. There is also a difference in the raw material economy at Sibudu, where knappers preferentially retouched and curated non-local tool stones.

Klein Kliphuis rockshelter (KKH) lies in the Western Cape of South Africa, approximately 200 km north of Cape Town and 70 km inland of the current coastline. The relevant assemblages of the “Early post-HP” derive from spits Dv and Dvi1-7 and date to ∼58 ka [40], [59]. We summarize the descriptions of these lithic assemblages by Mackay [56], [59].

Silcrete, quartz and quartzite are local raw materials and account for almost all artifacts, with rare non-local hornfels (<1%). Quartzite constitute the most common raw material overall, but there are marked changes in the procurement of tool stones. Blades amount to 10–20% of blanks with the rest being flakes of around 30–40 mm length (see in [56]; Fig. 8). Facetted platforms are frequent (16–41%) and the knappers employed Levallois, radial, platform and bipolar core reduction methods. KKH features many large cores (14–259 g), with few intensively reduced or exhausted specimens. The blades have a mean platform thickness of ∼5 mm, EPAs of 82° and are often facetted (33%). Retouched specimens constitute 6% of all artifacts >25 mm (A. Mackay, pers. comment). Unifacial points are the most common formal implements, followed by scrapers. The actual number of unifacial points numbers, however, is low (*cf*. [56], Fig. 5): no units yielded more than five unifacial points and five spits exhibit only one or none. Backed tools occur in the earliest layers of the “post-HP” (Dvi6-7) as well as six bilaterally backed points. The high number of lithic products suggest intensive occupations and knapping activities. Mackay [120] also provides mean edge length to mass ratios of 28.65 for layers DV-Dvi7, fluctuating between 20–40.

The “Early post-HP” at KKH resembles the Sibudan assemblages BM-BSP in terms of blank production, core reduction and core preparation. While there is no information on the production of convergent flakes, the size of the flakes and the proportions of blades to flakes are also similar. Unifacial points constitute the most frequent formal tool type at KKH, but their absolute number is very low with a diminished diversity in forms compared to the six Sibudan assemblages. The unifacial points depicted (see in [59], Fig. 8) resemble Tongatis. Comparable pieces to NBTs, Ndwedwes or ACTs are not presented. Average values of flaking efficiency at Sibudu fall below the range of KKH, indicting a less efficient use of raw materials. Interestingly, the majority of cores at Sibudu is heavily reduced, which is not the case for KKH. The lack of non-local artifacts at KKH can best be explained by the local availability of high-quality lithic raw material. Based on the values for platform thickness, blade production proceeded by internal percussion, but the kind of hammer used remains unclear. A conclusive evaluation will need to include a more detailed assessment of the knapping technique for blades and flakes, a technological analysis of the blanks produced, and the economy and reduction sequences of raw materials.

Diepkloof Rock Shelter (DRS) lies around 15 km inland from the Altantic Ocean and yielded a thick stratigraphic sequence with frequent and intense occupations during the “post-HP” that compare well with Sibudu [18]. Porraz *et al.* [62] provide a short characterization of the lithic assemblages Danny to Claude (n = 1289, >20 mm), which are dated to 52+/−5 ka [20] and 55.4+/−2.0 ka [40].

The knappers used mainly silcrete, quartzite and quartz, with non-local raw materials amounting to ca. 50% of the assemblages. The majority of blanks are flakes (66%), followed by blades (19%), bladelets (8%) and few convergent flakes (3%). Core reduction is characterized by blade products, including HP-type debitage. Knappers produced blades with irregular forms by internal percussion using hard stone hammers. Flakes are morphologically variable and show unidirectional and centripetal dorsal negatives with little platform preparation. Retouched forms are frequent (14%). Scrapers in various reduction degrees constitute the most frequent tool form (27%), followed by unifacial points (14%). Some of the points show short triangular ends that are comparable to the Tongatis of the Sibudan (see in [62], Fig. 11: 7–9). Other tool forms include denticulates and notches (15%), burins (6%), truncated pieces (5%), backed pieces (4%) and splintered pieces (4%), and end scrapers (2%).

The provisioning with local and non-local raw materials, the production of flakes and blades, the coexistence of different core reduction methods and an emphasis on the distal reduction sequence reflect similarities between the “post-HP” at Sibudu and DRS. However, Porraz *et al.* [62] note that there are important typological and technological differences between these assemblages, such as the lack of unifacial point categories other than the Tongatis and the absence of NBTs. They conclude that the “post-HP” at DRS should thus not be subsumed under the “Sibudu technocomplex” (*sensu* [9]). To these observations, we add that the production of convergent flakes only plays a negligible role at DRS and blades in the Sibudan assemblages BM-BSP are more regular and produced by soft stone hammers. Discoid technology and more frequent core preparation also distinguish these layers from Danny to Claude at DRS.

Our site by site comparisons demonstrate that the Sibudan assemblages that we have studied so far show several parallels in terms of technology, techno-economy and typology to other sites dating to early MIS 3. But there are also important differences in these domains. In particular, the abundance of unifacial points – and tools in general –, the clear patterning of production cycles and reduction histories for specific tool classes (e.g. Tongatis, *sensu* [63]), the use of a soft stone hammer to produce blades, the frequent manufacture of convergent flakes and the co-existence of several core reduction methods, including the discoid method, distinguish the Sibudan from most of these assemblages. We see two potential explanations for the observed patterns. First, the lithic assemblages BM-BSP could be interpreted as a special, site-specific case of the “post-HP” due to particular environmental circumstances, patterns of site use, mobility patterns or raw material availability. As an alternative explanation, our findings can be interpreted as supporting the working hypothesis by Conard *et al.* [63] that the lithic assemblages dated to ∼58 ka at Sibudu yield a new signal of the early “post-HP” that can be attributed to a novel cultural-technological unit, the Sibudan.

We support the latter interpretation, as we made great efforts to compare the assemblages at Sibudu to sites that are as similar as possible in terms of dating, type of site occupation, raw materials, geographical an environmental parameters. Sibudu and its occupation sequence after the HP are not exceptional with regards to these characteristics. All assemblages that we have compared derive from similar timeframes, feature raw materials of high and low flaking quality, show all stages of the lithic reduction sequence and derive from sites with repeated and intensive occupations similar to residential camps. Furthermore, the six studied Sibudan assemblages share several features with other “post-HP” assemblages, especially with the nearby sites URS and RCC, and are thus not an entirely isolated phenomenon. The perceived uniqueness of the techno-typological signal could also be attributed to the fact, that the Late Pleistocene MSA lithic technology of eastern South Africa is poorly documented, with few sites available for comparison. More detailed information on the lithic technology of URS and RCC, especially for aspects that we could not yet compare, might reveal that they should be included within the Sibudan. In conclusion, we view the Sibudan as a working model that can help to organize part of the cultural sequence of the MSA during MIS 3. Based on the long excavation history, the thick and high-resolution stratigraphy and the outstanding preservation of materials, Sibudu is ideally suited to serve as a type site and reference point for further comparisons (see also [9], [63]).

In view of the current data basis, the Sibudan appears to be a phenomenon during early MIS 3 which does not cover the entire period following the HP in terms of geography and chronology. Our comparisons have revealed several techno-typological parallels to sites from the eastern part of southern Africa but more pronounced differences to localities from the Southern and Western Cape. We want to emphasize, however, that the results and comparisons described here reflect work in progress. For now, we presented technological, techno-functional, techno-economic and typological data for six Sibudan lithic assemblages (BM-BSP) that date to ∼58 ka and provided preliminary comparisons with other sites. Using these data, researchers can perform additional comparisons with assemblages post-dating the HP, test the utility of the Sibudan as a cultural-taxonomic unit and critically examine its spatio-temporal range. Regarding our own work at Sibudu, there are still many layers of the depositional sequence following the HP that need to be analyzed. The Tübingen fieldwork at Sibudu is ongoing with the aim to excavate the entire sequence that follows the HP in the coming years (see Figure 1). We expect to observe still greater variation in the strata dated to ∼58 ka that have not yet been excavated by our team. The study of this variability can document patterns of short-term cultural behavior within the Sibudan. Characterizing the full range of variation will also represent an essential next step in testing and refining the ideas presented here.

## Conclusion

The Late Pleistocene cultural sequence at Sibudu that we have studied here exhibits a distinct technological signal of modern humans living during the later MSA in the eastern part of South Africa. We were able to define key elements that characterize the lithic assemblages and document technological variability within a high-resolution stratigraphy. The markers that unite these assemblages occur in several independent technological and typological domains even though they differ in sample size and reduction intensity. Comparisons with other assemblages from southern Africa that post-date the HP demonstrate several techno-typological parallels, particularly with the geographically closest sites Rose Cottage Cave and Umhlatuzana. Having said that, the Sibudan assemblages BM-BSP yield a so-far unique combination of technological, typological and techno-economic characteristics. These results support the use of the Sibudan (*sensu* [63]) as a concept that can serve as a starting point for comparisons with other MSA assemblages of this timeframe. Further research on local, regional and sub-continental scales is necessary and will help to assess the spatio-temporal distribution of the Sibudan. This work should evaluate whether the Sibudan is confined to the eastern part of southern Africa during early MIS 3 or covers a broader geographical and chronological range. These studies will also help to define the place of the Sibudan in the taxonomic hierarchy (e.g. [9], [143], [144]).

The findings that we have presented here, alongside recent studies by other researchers [54], [56], [57], [59], [60], [62], demonstrate the need to intensify research on periods that follow the SB and HP. From our analysis, we conclude that there is no reason to denote the technology of people living after the HP as “unsophisticated”, “conventional” or a “dark age”. Rather it seems to us that the lack of attention and detailed analyses devoted to this phase of the MSA resulted in a distorted picture. The results from the Sibudan assemblages BM-BSP refute these assertions by demonstrating that the knappers possessed a highly structured and sophisticated lithic technology. These findings are consistent with recent lithic studies at Diepkloof [62], Klasies River [60], Rose Cottage Cave [35] and Klein Kliphuis [56], suggesting that with an increased knowledge of this time frame, we gain a more realistic picture of spatial and temporal patterning of technological variability and cultural evolution of modern humans during the MSA of southern Africa.

Finally we stress that we do not see defining the Sibudan as a movement toward creating a rigid cultural taxon, but as part of a process of inquiry and a step toward gaining a better understanding of the cultural dynamics of the MSA. Here we follow the arguments made by Brew [154] decades ago and view cultural taxonomy as a tool to help archaeologists answer questions about the past and as a means of organizing our ideas about the past. Like Brew, we are not striving to create a single, ideal taxonomy that is universally valid, for such a goal is illusory and ultimately futile. Instead we are working to identify the cultural variability at Sibudu as part of the process of characterizing the behavioral patterning within the MSA. The critical assessment of the Sibudan may or may not confirm the usefulness of this approach, but, by presenting these results, we intend to further our understanding of the cultural dynamics of the MSA and thereby provide new insights into the behavioral patterns of modern humans in southern Africa shortly before the main expansion of our species across the Old World. Since the study of this phase of the MSA has been neglected in the past, we hope to have shown that the period following the HP does warrant our close attention. The intense research in recent decades in southern Africa makes the subcontinent a suitable region for developing more precise models of cultural evolution during the MSA. Only through detailed studies of multiple regions within southern Africa and Africa as a whole will we have any chance of determining what role, if any, the cultural evolution in southern Africa played in the successful expansion of our species around the globe.
